# A New Story of the Three Magi: Scaffolding Proteins and lncRNA Suppressors of Cancer

**DOI:** 10.3390/cancers13174264

**Published:** 2021-08-24

**Authors:** Larissa Kotelevets, Eric Chastre

**Affiliations:** Sorbonne Université, INSERM, UMR_S938, Centre de Recherche Saint-Antoine (CRSA), 75012 Paris, France

**Keywords:** PDZ domain, tumor suppressor, PTEN, Wnt signaling, Hippo signaling, cell junctions, lncRNA, MAGI1, MAGI2, MAGI3

## Abstract

**Simple Summary:**

MAGI1, 2, and 3 belong to a subgroup of the MAGUK family of scaffolding proteins, and are comprised of 6 PDZ domains, 2 WW domains, and 1 GUK domain. MAGIs associate with cell surface receptors, junctional complexes, and interact selectively with a wide range of effectors, including the PTEN tumor suppressor, the β-catenin, and YAP1 proto-oncogenes. The regulation of the PI3K/AKT, the Wnt, and the Hippo signaling pathways, on the one hand, the downmodulation of MAGIs in various types of cancers, and its physiopathological significance, on the other, make these scaffolding proteins considered to be tumor suppressors. Interestingly, *MAGI1* and *MAGI2* genetic loci generate a series of long non-coding RNAs (lncRNAs) that act as promoters or suppressors of tumors in a tissue-dependent manner by sponging some sets of miRNAs or by regulating epigenetic processes. This review details current knowledge of paths followed by the three MAGIs to control carcinogenesis.

**Abstract:**

Scaffolding molecules exert a critical role in orchestrating cellular response through the spatiotemporal assembly of effector proteins as signalosomes. By increasing the efficiency and selectivity of intracellular signaling, these molecules can exert (anti/pro)oncogenic activities. As an archetype of scaffolding proteins with tumor suppressor property, the present review focuses on MAGI1, 2, and 3 (membrane-associated guanylate kinase inverted), a subgroup of the MAGUK protein family, that mediate networks involving receptors, junctional complexes, signaling molecules, and the cytoskeleton. MAGI1, 2, and 3 are comprised of 6 PDZ domains, 2 WW domains, and 1 GUK domain. These 9 protein binding modules allow selective interactions with a wide range of effectors, including the PTEN tumor suppressor, the β-catenin and YAP1 proto-oncogenes, and the regulation of the PI3K/AKT, the Wnt, and the Hippo signaling pathways. The frequent downmodulation of MAGIs in various human malignancies makes these scaffolding molecules and their ligands putative therapeutic targets. Interestingly, *MAGI1* and *MAGI2* genetic loci generate a series of long non-coding RNAs that act as a tumor promoter or suppressor in a tissue-dependent manner, by selectively sponging some miRNAs or by regulating epigenetic processes. Here, we discuss the different paths followed by the three MAGIs to control carcinogenesis.

## 1. Introduction

The cellular response to external stimuli e.g., hormones, growth factor, cell–cell contact, stress requires the spatio-temporal integration of the several signaling pathways to produce an adapted biological response in terms of metabolism, proliferation, differentiation, cell–cell interaction, migration, or cell death. These processes are orchestrated at specific sites by scaffolding proteins that serve as the backbone for supramolecular signaling complexes termed signalosomes [1,2,3,4]. Scaffolding proteins allow the local and subtle tuning of effectors in the specific subcellular domain, the stimulation of distinct pathways simultaneously, or the coordination of positive and negative signals to impede or synergize the cellular response. These adaptors might act on their binding partners by triggering conformation changes, by favoring posttranslational modifications, by enabling or impairing the interaction with their upstream or downstream effectors, or by affecting protein stability or proteolytic degradation.

At the molecular level, scaffolding proteins can be considered as the modular assembly of domains able to interact with other proteins, nucleic acids (RNA, DNA), or lipids at the plasma membrane. More than 80 distinct protein interaction modules for intracellular proteins were referenced in Tony Pawson’s website in 2016 (http://pawsonlab.mshri.on.ca/index13a5.html accessed on 12 April 2021). Some of these domains are highly selective and have few targets, whereas others might interact with motifs present in a broad range of proteins, e.g., sequences rich in proline amino residues for SH3 and WW domains, the last 4–7 amino acids at the C-terminus of target proteins for PDZ domains (see below), a phosphorylated tyrosine residue for SH2 and PTB domains, phosphorylated serine or threonine residues for 14-3-3 domain. It is worth noting that the sequence inside or surrounding these recognition motifs contributes to the relative selectivity towards the interaction with these domains. Thus, each member of these families might selectively interact with a subset of target proteins. Scaffolding molecules are constituted by the layout of distinct protein interaction modules, e.g., SH3, PDZ for MAGUK family of proteins, or by the multiplicity of the same interaction domain, e.g., 13 PDZ domains for MUP1.

PDZ domains are composed of about 80–90 amino acids and form a classical two α-helical/six β-strand structure (for detailed structures, see [5,6,7,8]). Proteins with PDZ domain are involved in the anchoring of cell surface receptors and cell adhesion molecules to the actin cytoskeleton, in receptor desensitization, in cytoskeleton remodeling, in signal transduction, and proved to exert a critical role in regulating key biological processes, including cell–cell interaction, cell polarization, migration, proliferation, and survival [9,10,11,12,13]. The letters PDZ stand for the original identification of this domain in three unrelated proteins, i.e., postsynaptic density 95 (PSD95), disc large 1 (DLG1), and zonula occludens 1 (ZO1). Recent analysis suggests the existence of 154 distinct human proteins (excluding splice variants) containing 272 unique PDZ domains [8]. These globular domains mainly interact with the carboxy-terminus of partner proteins, but also with internal motifs and likewise, they can also oligomerize. About 40% of PDZ domains proved also to bind phosphatidylinositides and cholesterol [11,14,15]. Originally, PDZ domains were divided into three classes based on the last amino acids of the C-terminal target proteins. Class I PDZ domains recognize the motif sequence X-[S/T]-X-φ (X = any amino acid, φ = hydrophobic residues I, L, V, or F), Class II domains recognize X-φ-X-φ, and Class III domains recognize X-D/E-X-φ [7,11,16,17,18]. Further subdivisions have been proposed later on [8,19].

One single PDZ domain may interact with many different PDZ binding motifs. Conversely, one PDZ binding motif may be targeted by distinct PDZ domains. For instance, the C-terminus of β-catenin can interact with more than 25 proteins with the PDZ domain, among them NHERF1, DVL1, SAP97, and TIAM1, and MAGI1B via its domain PDZ5 [20,21,22]. On the other hand, the PDZ5 domain of MAGI1B can also interact with LRP2 (LDL receptor-related protein 2, megalin), α-actinin-4, FCHSD2 (FCH and double SH3 domains, carom), TRIP6 (thyroid hormone interacting protein 6, zyxin related protein 1), SDK1 (sidekick cell adhesion molecule 1) and SHC (SHC transforming protein) [23,24,25,26,27,28]. It is noteworthy that the β-catenin C-terminus does not bind to the PDZ2 nor the PDZ4 domains of MAGI1, highlighting the selectivity of PDZ domains for their target molecules [22].

In line with their ability to regulate various transduction pathways, many scaffolding proteins with PDZ domains, including syntenin, disheveled, TIAM1, GIPC, NHERF2, SCRIB, DLG, and MAGIs have been implicated positively or negatively in different steps of cancer development and dissemination. In the present review, we focused on the three MAGIs, as an archetype of scaffolding molecule with antitumor activity.

## 2. Implication of MAGI Molecular Scaffolds in the Control of Carcinogenesis

### 2.1. MAGI Structure

Among the scaffolding molecules with PDZ domains, the membrane-associated guanylate kinases (MAGUKs) are a superfamily of proteins characterized by the inclusion of one or three PDZ domains at the N-terminus, followed by one SH3 domain and then the guanylate kinase (GUK) domain. This GUK domain is catalytically inactive due to the absence of the P-loop that binds ATP. Nevertheless, it allows protein interaction, mainly with cytoskeleton proteins and effectors of signal transduction. Many MAGUK proteins contain other modules, e.g., homologous of CaMKII, WW, or L27 domains [11]. PSD95, DLG1, and ZO1 that serve as an acronym for “PDZ” are members of this family of proteins. 

**Figure 1:**
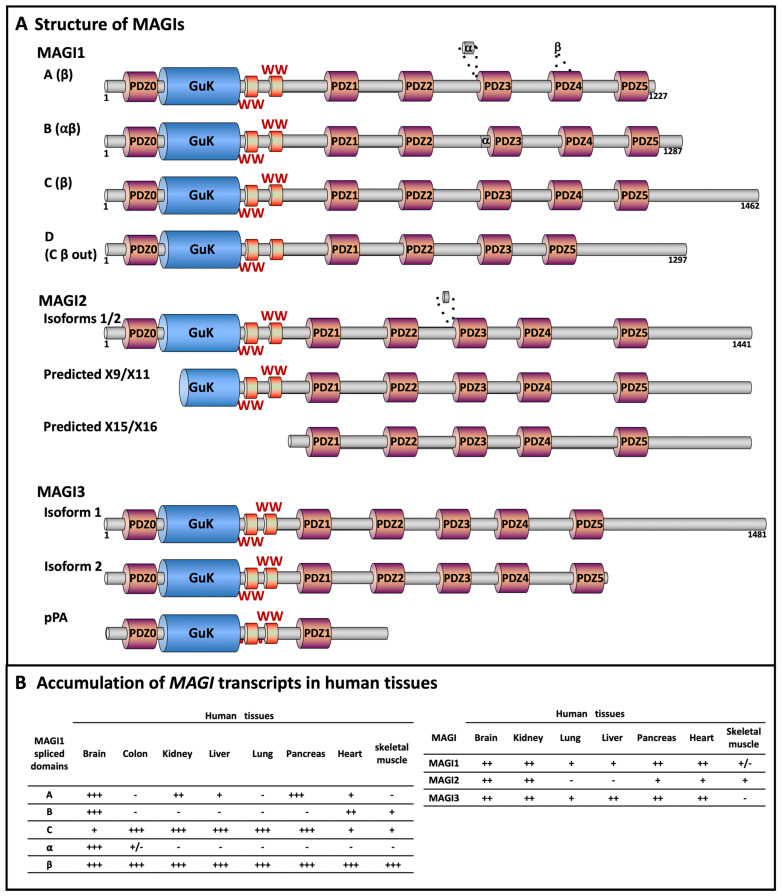
Primary structure of MAGI scaffolding molecules. (**A**) MAGI1, 2, and 3 are comprised of 6 PDZ domains, 2 WW domains, and 1 GUK domain. *MAGI* transcripts are subjected to posttranscriptional modifications leading to the emergence of several variants. PDZ: PDZ (PSD95, DLG1, and ZO1) domain, numbered from 0 to 5; GuK: guanylate kinase domain; WW: WW domain (also termed rsp5-domain or WWP repeating motif). MAGI1. The isoforms MAGI1A, B, and C result from alternative splicing downstream the sequence encoding PDZ5, the related frameshifts generating unique C termini. The C terminus of MAGI1C contains potential nuclear targeting signals. Two internal splice variants have been further evidenced, these include an in-frame insertion of 28 amino-acid residues upstream PDZ3 domain (α domain). The 2nd splicing involves a region termed β, encoding much of PDZ4 [23,32]. Four different arrangements of the β region were identified, each encoding an intact PDZ4 which might exhibit distinct ligand binding properties. Splicing out the β domain results in the depletion of a functional PDZ4 domain, and in an isoform encompassing 5 PDZ domains. A MAGI1C variant with splice out of β region and part of the inter-domain connecting PDZ3 and PDZ4 was identified in the human cervix and designed MAGI1D [39]. The structure of the MAGI1 splice variants displayed in panel A is based on the initial nomenclature and sequences from Laura et al. (GenBank AF401655, AF401654, and AF401656) and van Doorslaer et al. [32,39]. Other MAGI1 predicted variants are not represented. MAGI2. The MAGI2 isoform 1 differs from isoform 2 by in-frame insertion of 14 amino acid residues in the inter-domain linker connecting PDZ2 and PDZ3 (alternative splice schematized by the disc and the dashed lines). Some of the predicted MAGI2 isoforms arising from alternative translation initiation sites are also displayed. MAGI3. The MAGI3 isoform 2 results from an alternate splice site at the 5’ end of the last coding exon, generating a shorter and distinct C-terminus. MAGI3pPA originates from the use of a cryptic intronic poly(A) signal. (**B**) Tissular distribution of *MAGI* transcripts. Relative abundance of the transcripts encoding MAGI1 isoforms (left panel) [32], and MAGI1, 2, and 3 (right panel) in human tissues, as evaluated by RT-PCR and northern blot [31,43,44,45,46]. Note that MAGI1 is also designated: atrophin-1-interacting protein 3 (AIP-3), BAI1-associated protein 1 (BAP-1), membrane-associated guanylate kinase inverted 1 (MAGI-1), trinucleotide repeat-containing gene 19 protein, WW domain-containing protein 3 (WWP3); MAGI2 is also named: atrophin-1-interacting protein 1 (AIP-1), atrophin-1-interacting protein A, synaptic scaffolding molecule (S-SCAM), membrane-associated guanylate kinase inverted 2 (MAGI-2); and MAGI3: membrane-associated guanylate kinase inverted 3 (MAGI-3).

The three membrane-associated guanylate kinase inverted (MAGI) proteins constitute a subgroup of MAGUK proteins. They are characterized by an inverted arrangement: the GuK domain is at the NH2 terminus, the SH3 domain is replaced by two WW domains, and follows five PDZ domains –numbered 1 to 5- [29,30]. A more recent analysis of the MAGI structure allowed to identify an additional PDZ domain above the GUK domain that was designed PDZ0 (Figure 1A) [31,32]. Overall, the primary sequence of MAGI3 is more similar to MAGI2 than to MAGI1 [8,33,34]. MAGI1 and MAGI3 are ubiquitous, whereas MAGI2 is mainly expressed in the brain and the renal glomeruli (podocytes), followed by skeletal muscle, pancreas, ovary, and heart (Figure 1). These scaffolding molecules are mainly located at junctional complexes, including tight and adherens junctions [21,22,29,32,35,36,37,38]. Several splice variants of MAGIs have been evidenced and proved to be expressed in a tissue-dependent manner (Figure 1B) [23,29,32,39]

Two MAGI2 variants termed (MAGI2α and MAGI2β) depleted of PDZ0 and part of the GUK domain as the result of alternative translational initiation sites have been also identified in the rat brain [40]. Such variants in human tissues have also been predicted from genomic analysis and expressed sequence tags. 

The premature polyadenylation of *MAGI1* and *MAGI3* transcripts have been evidenced in human small intestinal neuroendocrine tumors and breast cancers, respectively [41,42]. The use of a polyA site in the 2nd intron of MAGI1 upstream the sequence encoding the GuK domain leads to the expression of an mRNA lacking 21 exons in the 3-end, whereas the premature polyadenylation of MAGI3 produces a truncated protein depleted of PDZ2-PDZ5 [41,42]. 

### 2.2. MAGI Molecular Partners

**Figure 2:**
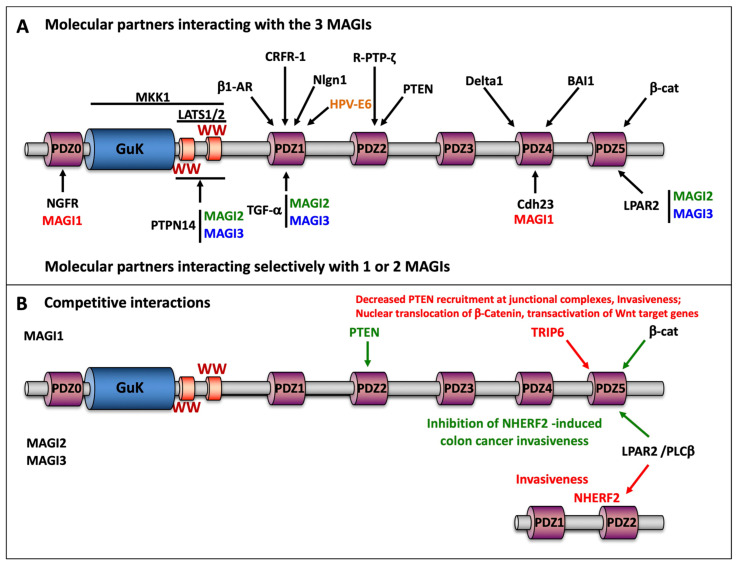
Molecular partners of MAGI scaffolding molecules. (**A**) The scheme displays the selective interaction of MAGIs with molecular partners that are either shared by these three scaffolding molecules or restricted to a subset. For easy viewing, MAGI1, 2, and 3 are displayed in red, green, and blue fonts, respectively. The cellular molecular partners are in black font and the viral oncoprotein E6 in orange font. (**B**) MAGIs can compete with other scaffolding molecules, e.g., NHERF2 to interact with the same molecular target (MAGI2, 3). Conversely, some molecular partners of MAGIs can compete for interacting with the same binding module, e.g., β–catenin vs. TRIP6 (MAGI1). These competitions trigger distinct signaling pathways and cell fate. The prooncogenic pathways are depicted in red, the tumor suppressor pathways in green. β1-AR: beta-1 adrenergic receptor; BAI1: brain-specific angiogenesis inhibitor 1/ adhesion G protein-coupled receptor B1; Cdh23: cadherin 23; CRFR-1: corticotropin-releasing hormone receptor 1; β–cat: catenin beta-1; Delta1: delta-like protein 1; LATS1/2: large tumor suppressor homolog 1/2; LPAR2: lysophosphatidic acid receptor 2; MKK1: mitogen-activated protein kinase kinase 1; NGFR: low-affinity nerve growth factor receptor; NHERF2: Na(+)/H(+) exchange regulatory cofactor NHE-RF2; Nlgn1: Neuroligin-1; PLC β: 1- phosphatidylinositol 4,5-bisphosphate phosphodiesterase beta; PTEN: phosphatase and tensin homolog; PTPN14: protein tyrosine phosphatase non-receptor type 14; R-PTP-ζ: receptor-type tyrosine-protein phosphatase zeta; TGF-α: (pro)transforming growth factor-alpha; TRIP6: thyroid receptor-interacting protein 6; HPV-E6: protein E6 human papillomavirus.

Many ligands of MAGIs have been identified, including KRAS, NET1, PTEN, β-catenin, NMDA receptors, neuroligin, BAI-1, megalin, RA-GEF-1, activin type II receptor (Table 1). These ligands may compete with each other for interacting with the same MAGI binding module (Figure 2). Some of these interactions are shared by the three MAGIs (e.g., PTEN, β-catenin), others seem to be restricted to a subset of these scaffolding molecules (e.g., the nerve growth factor receptor interacts with MAGI1, but not with MAGI2 or MAGI3; Figure 2, Table 1). Thus, it seems difficult to delineate a specific biological activity for each MAGI, although some selective tissue expression has allowed establishing some peculiar physiological role. In this context, the constitutive knockout of MAGI2 in mice leads to podocyte loss, diffuse glomerular extracapillary epithelial cell proliferation, and renal failure [47]. 

The 9 binding modules of MAGIs allow the spatiotemporal organization of signalosomes and the subcellular targeting/confinement of effector proteins (e.g., PTEN or β-catenin at adherens junctions), as well as gathering effectors of the same pathway (e.g., the Wnt receptor frizzled 4/Vang-like protein 2) or with antagonistic activities (e.g., ERBB4 tyrosine kinase receptor/tyrosine phosphatase R-PTP-zeta; Rho guanine nucleotide exchange factors NET1/Rho GTPase activating protein ARHGAP6) for the fine-tuning of downstream signaling pathways (Figure 2).

### 2.3. MAGIs in Cancer

#### 2.3.1. Genetic Alteration, Silencing

The frequent genetic alteration and downregulation of MAGIs at transcripts and protein levels in many types of cancers, including breast, prostate, liver, stomach, colon, and cervix offer a valuable insight into the implication of these scaffolding proteins in the control of carcinogenesis (Table 2). 

The promoter methylation and the silencing of *MAGI1* have been reported in anaplastic thyroid cancer and acute lymphocytic leukemia [121,122]. Methylation of multiple CpG islands of *MAGI1* promoter is associated with a worse overall survival of patients with lymphocytic leukemia [122]. MAGI-1 expression proved also to be frequently downregulated at mRNA and protein levels in human T-cell leukemia cell lines [123]. Methylation of the *MAGI2* gene was evidenced in cervical, gastric, endometrial, ovarian, and breast cancers [124,125,126,127,128]. In the cervix carcinoma HeLa cell line, this hypermethylation was attributed to the downregulation of TET1 -involved in DNA demethylation via the oxidation of 5-methylcytosine- by the HOTAIR lncRNA [129]. 

Some genomic rearrangements of *MAGI2* have also been evidenced in melanoma cell lines and prostate cancers leading to in-frame deleted transcript with an unknown biological significance, and to *MAGI2* invalidation, respectively [130,131]. 

A rearrangement between *MAGI3* and *AKT3* genes caused by chromosome 1 inversion, has been identified in triple-negative breast cancer (lacking estrogen and progesterone receptors and ERBB2 expression). The resulting chimeric protein combines MAGI3 truncated upstream PDZ1, fused with AKT3 [132]. Nevertheless, the overall prevalence of this constitutively active protein kinase seems to be below 1% of tumors [133,134]. 

*MAGI1* mutation in early gastric cancer and adjacent mucosa is associated with an increased risk of developing metachronous gastric cancer following curative endoscopic submucosal dissection [135]. Overall, missense mutations of *MAGI* genes are identified in 16–20% of human cancers, non-sense mutation being in the range of 1–2% (Cosmic V94, URL http://cancer.sanger.ac.uk/cosmic/, released 28 May 2021, accessed on 22 July 2021, Table 2).

Another level of MAGI dysregulation relates to the targeting of the corresponding transcripts by a series of microRNAs (Table 2). *MAGI1* mRNAs are down-regulated by miR-486-5p in the human erythroid leukemia K562 cell line, leading to decreased RA-GEF-1 /Rap1A signaling pathway and inhibition of erythroid differentiation [136]. In renal cell carcinoma, a low *MAGI1* expression level, as a result of c-Myb-induced miR-520h transactivation, is associated with a poor prognosis [137]. *MAGI2* is targeted by a series of miRNA including miR-629-5p, miR-487a, miR101, and miR-134/487b/655 cluster [138,139,140,141]. In breast cancers, miR-487a was positively correlated with lymph nodes metastasis and negatively correlated with the expression of *MAGI2*. At the mechanistic level, miR-487a is transactivated by the TGF-β /NF𝜅B signaling pathways and promotes epithelial–mesenchymal transition (EMT) and invasiveness via targeting MAGI2/ PTEN axis [139]. In these cancers, miR-101 impedes the same pathway to enable estrogen-independent growth [140]. MiR-27a-3p functions as an oncogenic miRNA in breast cancer cells themselves, but also redesigns the tumor microenvironment. Accordingly, the release of this oncomiR in exosomes from breast cancer cells downregulates MAGI2 in macrophages, leading to upregulation of PDL1 and immune escape of cancer cells [142]. In lung carcinoma cell lines, miR-134/487b/655 cluster regulates TGF-β-induced EMT and drug resistance to gefitinib by targeting *MAGI2* [141]. 

As regards *MAGI3*, its downregulation by oncomiR-34c-3p enhances β-catenin/ c-Myc -induced glycolytic gene expression/ Warburg effect and promotes the growth of human hepatocellular carcinoma cell lines in vitro and in vivo [143].

MAGIs are also subjected to posttranslational modifications, including phosphorylation, SUMOylation, and cleavage by caspase 3 and 7 (Table 1), whose regulation, biological impact, and pathological implications remain are so far poorly documented.

#### 2.3.2. MAGIs Expression and Prognosis

As mentioned above, the accumulation of MAGI transcripts and proteins is proven to be associated with prognosis in various human malignancies (Table 3). In renal, liver, and gastric cancers and in lymphocytic leukemia, MAGI1 downregulation is associated with a poor prognosis (Table 3) [122,137,145,146]. In gliomas, MAGI1 is downregulated at both transcript and protein levels, and this expression is negatively correlated with the WHO grade of the tumors [56]. In estrogen receptor-positive (ER+)/human epidermal growth factor receptor 2 negatives (HER2−) breast cancers, MAGI1 is considered a tumor suppressor [88,147]. In this subtype of tumors, MAGI1 expression is positively correlated with the accumulation of the transcript encoding estrogen receptor 1, the luminal fate transcription factors GATA3 and FOXA1 levels, and a lower risk of relapse, whereas low MAGI1 levels correlate with more aggressive phenotype and worse prognosis. It turns out that MAGI1 and estrogen receptors mutually regulate each other. On the one hand, MAGI1 silencing in the MCF7 breast cancer cell line decreases *ESR1* mRNA and ERα protein levels, activates PI3K/Wnt signaling, promotes cell proliferation, and reduces apoptosis and epithelial differentiation. On the other hand, estradiol treatment of the ER+ MCF7 cell line upregulates *MAGI1* mRNA levels [147]. In the experimental model of metastasis, MAGI1 downregulation triggers the metastatic phenotype of the mouse mammary 67NR (ER+) cells, whereas its ectopic expression impairs the metastatic properties of the 4T1 (ER−) cells. MAGI1 expression negatively correlates with the inflammatory status of breast tumors and is downregulated by the prostaglandin E2/ COX-2 axis. These observations are in line with another study demonstrating that Cox-2 inhibitor upregulates *MAGI1* in human colon cancer cell lines [148]. 

As regards MAGI2, conflicting observations have been reported in prostate cancers (Table 3). Cao et al. found a decreased accumulation of *MAGI2* transcripts in prostate cancer as compared to control tissue, whereas the immunostaining of MAGI2 was unaffected [149]. They also reported that high MAGI2 expression is associated with longer disease-free survival. Similarly, Mahdian et al. reported a decreased accumulation of *MAGI2* transcripts in prostate cancer [150]. In contrast, other studies evidenced increased MAGI2 immunostaining in high-grade prostatic intraepithelial neoplasia (HGPIN) and tumor, as compared with control tissue [151,152,153,154,155], with a decreased expression during progression from HGPIN to adenocarcinoma of low to high Gleason score [151]. Nevertheless, it is noteworthy that *MAGI2* mutations are found at a relatively high incidence (26%) in these cancers (Cosmic V94, URL http://cancer.sanger.ac.uk/cosmic/, released 28 May 2021, accessed on 22 July 2021). In multiple myeloma, *MAGI2* is overexpressed and is significantly associated with unfavorable patient outcomes [156]. A super-enhancer (large clusters of enhancers) in the *MAGI2* gene is a driver of oncogenic phenotype in multiple myeloma and is further accentuated by binding the oncogenic MAF transcription factor [156]. Nevertheless, further studies are required to assign this phenotype to MAGI2 itself or other genetic elements, e.g., MAGI2-AS3 that shares the same promoter. In contrast, as stated above, *MAGI2* promoter is hypermethylated in many solid tumors, including cervical, gastric, endometrial, ovarian, and breast cancers. This hypermethylation and *MAGI2* silencing has been associated with a poor prognosis in gastric and breast cancers [125,128].

Concerning MAGI3, high levels of transcripts predict an excellent prognosis in ovarian cancers [157]. In human gliomas, MAGI3 is downregulated at both mRNA and protein levels, and this expression is negatively associated with tumor grade and prognosis [55]. Overexpression of *MAGI3* in the human U373 and LN229, and C6 rat glioma cell lines reduces cell proliferation, arrests the cell cycle, and inhibits migration [55]. 

The role of MAGIs in the control of carcinogenesis is further highlighted by human papillomaviruses (HPV) E6 viral oncoprotein-induced degradation. The high-risk types HPV16 and HPV18, are associated with cancer onset in humans, where the most common is cervical cancer. This pathogenesis is tightly linked to the combined action of E6 and E7 oncoproteins, which cooperate efficiently to immortalize human keratinocytes and whose sustained expression is required for maintaining the proliferative potential and to prolong the survival of tumor-derived cells. The E6 oncoprotein targets several proteins with the PDZ domain for degradation by the proteasome, among them MAGI scaffolding molecules through interaction with PDZ1 [109,158]. The use of E6 protein deficient in the PDZ binding motif allowed us to delineate the impact of the selective downregulation of protein with the PDZ domain in neoplastic progression. Single depletion of MAGI1, SCRIB, or PAR3 significantly but partially restored the tumorigenic ability of the E6 protein depleted of the PDZ binding motif [159]. On the other hand, the re-expression of a MAGI1 variant resistant to E6 targeting in HPV-positive cells increases recruitment of ZO-1 and PAR3 at cell–cell contact, represses cell proliferation, and triggers apoptosis [118]. Phylogenetic analysis of oncogenic and non-oncogenic papillomaviruses suggests that the ability of their E6 proteins to induce degradation of PDZ-proteins is an enabling phenotype towards oncogenicity [39]. Another level of regulation of E6 oncogenic properties depending on the HPV type relies on their differential phosphorylation by PKA or AKT, which, in turn, inhibits PDZ recognition [160].

#### 2.3.3. Signaling Pathways and Effector Systems Involved in the Tumor Suppressor Activities of MAGIs

**Figure 3:**
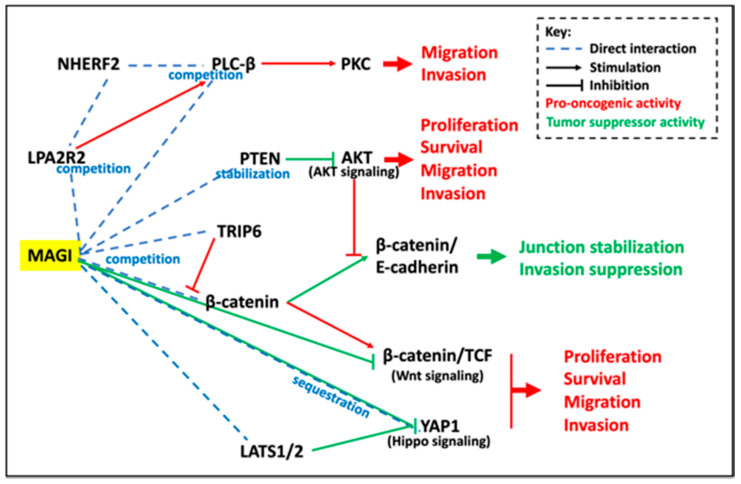
Schematic overview of the main MAGI interacting partners and effectors systems, and their biological impacts. The selective interaction of MAGIs with their molecular partners enables controlling concurrently several interconnected pathways involved in cell proliferation, survival, and dissemination. The recruitment of PTEN to the E-cadherin/β-catenin junctional complexes stabilizes PTEN, decreases both PI3K/AKT and Wnt signaling. MAGIs also act at many levels of the Hippo signaling pathways, and by competitive interaction overcome the activity of other signalosomes including NHERF. For details see the text.

As shown in Table 1 and Figure 2, MAGI proteins interact with a wide variety of effector proteins involved in the control of cell proliferation, apoptosis, or cell motility. Although not all of these interactions have so far been linked to MAGI antitumor activity (e.g., MAPK, Notch, Rho, Smad), some of them are clearly established (Figure 3) [164,165,166].

##### PTEN/PI3K/AKT Signaling Pathway

Phosphatase and tensin homolog (PTEN) is a tumor suppressor that represents one of the most prevalent targets for genetic alteration in human cancer. PTEN controls a broad range of physiopathological processes related to cell proliferation, differentiation, DNA/chromosome integrity, apoptosis, and invasiveness. PTEN dephosphorylates not only proteins but also phosphoinositides generated by phosphatidylinositol 3-kinase, thus counteracting the Akt signaling pathway [1,2]. PTEN exerts also some biological functions independently of its catalytic activity. PTEN contains a PDZ binding motif in its C-terminus and interacts with several proteins with PDZ domains including MAGI proteins through PDZ2 [22,96,97,98]. This interaction-impaired by PTEN C-terminus phosphorylation-, increases PTEN stability and enhances PTEN mediated downregulation of PI3K/AKT activity [22,31,56,96,123,139,140,167,168,169]. We demonstrated that the inhibition of the PI3K/ AKT pathways by the PTEN/MAGI1 signalosome exerts a critical role in restraining the invasive phenotype [22,170]. In this regard, MAGIs protein levels and PTEN accumulation and activity proved to be correlated [56,152,171,172]. This is of high importance since Alimonti et al. have demonstrated that, even a subtle reduction in PTEN level is sufficient to promote cancer susceptibility [173]. In this connection, celecoxib, a Cox-2 inhibitor upregulates MAGI1 in the human colon cancer SW480 and HCT116 cell lines, curtailing primary tumor growth and spontaneous lung metastasis in an orthotopic model of colorectal cancer [142]. Interestingly, celecoxib was also reported to promote the membrane translocation of PTEN and PTEN activity through deacetylation, leading to AKT inactivation [174]. One interesting point that was not addressed in this study, concerns the impact of MAGI1 expression on celecoxib-induced PTEN targeting to the plasma membrane. In the breast cancer MDA-MB-231 cell line, it has been reported that stimulation of PAR2 by tissue factor, transiently reduces PTEN interaction with MAGI2, this release being associated with enhanced PTEN activity [175]. 

MEK1 interacts with the GuK and WW domains of MAGIs proteins and proved to be necessary for PTEN recruitment at the plasma membrane and deactivation of the AKT pathway. This complex formation is independent of MEK1 kinase activity but requires MEK1 phosphorylation at T292 by ERK [94]. These interconnections between the RAF/MEK1/ERK and PI3K/AKT pathways suggest that RAF or MEK inhibitors might promote AKT activation and emphasize the requirement of combined targeting of these two pathways to circumvent the emergence of resistance to chemotherapies. 

##### Beta-Catenin/Wnt Signaling Pathways

Beta-catenin is a genuinely multifunctional protein, with two major cellular pools. It localizes (i) at the plasma membrane allowing the anchoring of cadherin cell–cell adhesion molecules to the actin cytoskeleton, and (ii) free in the cytosol or nucleus as an effector of the Wnt pathways that when disturbed is a major driver of cancer. Activation of the Wnt receptor frizzled allows accumulation and nuclear translocation of β-catenin, interaction with the TCF/LEF family of transcriptional factors, and the transactivation of target genes involved in cell proliferation, survival, and migration. In absence of Wnt ligands, β-catenin is maintained at a low level by the glycogen synthase kinase 3β (GSK3β). GSK3β forms a multimeric complex with APC, AXIN1, and β-catenin and phosphorylates the N-terminus of β-catenin, leading to its ubiquitination and degradation by the proteasome [176]. 

Beta-catenin does interact with MAGI PDZ5 at the plasma membrane [21,22,36,55]. This interaction strengthens junctional complexes (*Vide infra*) and prevents the nuclear translocation of β-catenin and the transactivation of Wnt target genes (Figure 3). In this context, MAGI3 knockdown in the PTEN defective U373 glioma cell line induces accumulation of the Wnt transcriptional targets cyclin D1 and Axin2 and enhances cell proliferation and migration [55]. Similarly, downregulation of MAGI1 in the PTEN defective U87MG and U251 glioblastoma cell lines enhances the accumulation of β-catenin, increases the expression of mesenchymal biomarkers, stimulates cell proliferation, and reduces cell apoptosis [56]. 

MAGIs might also control Wnt signaling through the PTEN/PI3K/AKT pathway. Accordingly, AKT phosphorylates β-catenin at Ser 552, which causes its dissociation from cell–cell contacts and accumulation in the cytoplasm and the nucleus, leading to β-catenin enhanced transcriptional activity and cancer cell invasiveness [177]. In contrast, the phosphorylation and inactivation of GSK3 by AKT do not seem to be involved in the control of the Wnt signaling. There may be different pools of GSK-3: one linked with Axin that is resistant to phosphorylation by Akt, and another pool that is regulated by AKT but did not appear to modulate β-catenin signaling [178,179]. 

Interestingly, in brain synapses, MAGI2 (S-SCAM) forms a complex with β-catenin and competes with GSK3 for AXIN binding and may protect β-catenin from degradation [89].

MAGI1 and MAGI3 are also involved in the non-canonical Wnt signaling cascade regulating planar cell polarity [67,68], including the stereociliary bundles of the cochlea [180]. MAGI3 forms a ternary complex with Frizzled-4 and Vangl2 (Vang planar cell polarity protein 2) via its PDZ1—suggesting MAGI3 oligomerisation—and potentiates JNK activation in a Rac1-dependent manner [68].

##### HIPPO Signaling Pathway

YAP1 (Yes1 Associated Transcriptional Regulator) is the critical regulatory target in the Hippo signaling pathway. Hippo pathway regulation of YAP1 mediates cellular responses to mechanical tension, extracellular ligands, energy, osmotic and hypoxic stress, and inflammation and tissue injury [181]. In the nucleus, YAP1 interacts with transcription factors including TEA domain family members (TEADS) that trigger the expression of target genes implicated in cell transformation, proliferation, invasion, and metastasis [182]. The Hippo pathway involves a kinase cascade, including the Ser/Thr kinases STK3/ STK4, that activates LATS1/2 in complex with their regulatory protein MOB1, which in turn phosphorylate and inactivate YAP1 oncoprotein through cytoplasmic sequestration by 14-3-3 proteins [183]. 

A role of MAGI3 in the control of YAP1-dependent transformation was recently evidenced in breast cancer, through the interaction of the C-terminus of the transcriptional regulator with the PDZ5 of the scaffolding molecule [42]. In the human mammary MDA-MB-231 cancer cells, the premature polyadenylation of MAGI3 produces a truncated protein depleted of PDZ2-PDZ5 (*MAGI3^pPA^*, Figure 1) that acts in a dominant-negative manner to prevent full-length MAGI3 from interacting and impairing YAP1 nuclear translocation (Figure 3) [42,90]. Silencing of *MAGI3^pPA^* transcripts and YAP1 activity markedly decreases the growth of MDA-MB-231 cancer cells xenografted in NOD/SCID mice, whereas ectopic expression of the variant promotes anchorage-independent growth of MCF10A-SV40 cells. This premature polyadenylation at a cryptic intronic poly(A) signal in MAGI3 identified in 7.5% of breast cancers -but not in tumor-adjacent control tissues- likely contributes to breast carcinogenesis. This variant is not related to mutations in the exon-intron boundaries but is associated with reduced N6-methyladenosine in *MAGI3* large internal exon [90]. The premature polyadenylation of *MAGI1* has also been reported, but its physiological significance remains unknown.

In line with MAGI3 control of the Hippo signaling pathway, an RNA-interference screen revealed that *MAGI1* silencing decreases YAP1 phosphorylation, triggers its nuclear translocation, and the transactivation of a reporter gene [184]. The underlying mechanisms have not been investigated. Interestingly, using the proximity-dependent biotinylation identification technology (BioID) in HeLa and HEK293 cells, Couzens et al. reported the interaction of MAGI1 with LATS1, presumably via the WW domain [93]. MAGI2 and MAGI3 were also recently reported to interact with LATS1/2 as well as with other effectors of this signaling pathway via their WW domain tandems, including AMOT (angiomotin) and PTPN14 [34]. These tandems enhance the binding affinity and specificity for their targets [34]. Alternatively, MAGI proteins might indirectly control the YAP1 oncogenic pathway through the PTEN tumor suppressor. Accordingly, loss of PTEN phosphatase activity leads to LATS/MOB1 complex destabilization, decreased YAP1 phosphorylation and nuclear translocation, and promotes cell proliferation and migration of gastric cancer cells in vitro, and in vivo after xenografting in nude mice [185]. Furthermore, GSK3, which is inhibited by AKT, phosphorylates and triggers the degradation of the YAP1 related molecule TAZ [186]. Nevertheless, the regulation of YAP1 by MAGIs is seemingly complex. Accordingly, in endothelial cells, MAGI1 depletion markedly increases LATS1/2 at mRNA and protein levels leading to a reduction in YAP expression and endothelial permeability [106].

##### Junctional Complex Stabilizer, Invasion Suppressor Activity

Besides their critical role in the control of cell signaling, MAGI scaffolding proteins allow complex formation between a series of transmembrane proteins and actin-binding proteins at tight and adherens junctions (Figure 3). 

Tight junctions exert a critical role in the maintenance of epithelial barrier integrity [4,10,12,38]. Increased paracellular permeability of intestinal epithelium allows increased translocation of commensal bacteria from the gut lumen and the development of inflammation, a driver of colorectal cancer [4]. In this concern, it is worth noting that genetic variants of *MAG1*, *2*, and *3* have been associated with the development of inflammatory bowel diseases, including ulcerative colitis and Crohn’s disease [187,188,189,190,191]. These pathologies are at high risk in the subsequent development of colorectal cancers [4,192,193,194]. The role of MAGIs in the maintenance of tight junctions is further highlighted by the disruption of these junctions by viral oncoproteins that drive MAGIs to proteasomal degradation. Restoration of MAGI1 expression in the HPV-positive cervix cancer HeLa cell line induces cell growth arrest and apoptosis [118]. MAGI1 proves also to be a target of caspase-3 and -7 during the apoptotic process, MAGI1 cleavage contributes to cell junction disassembly, a key feature of apoptosis [103]. In endothelial cells, thrombin stimulates p90RSK that directly phosphorylates MAGI1 at Serine 741 and induces MAGI1 deSUMOylation at Lysine 931, the consecutive release of MAGI1 from adherens and tight junctions leading to endothelial permeability [106]. In this model, this effect was mimicked by MAGI1 down-regulation. The interaction of β-catenin with MAGI1 seems to be involved in the maintenance of tight junction integrity [20].

MAGIs act also as a suppressor of invasiveness through the stabilization of adherens junctions. We previously demonstrated that ectopic expression of MAGI1B enhanced the recruitment of PTEN to junctional complexes, promoted E-cadherin-dependent cell–cell aggregation, and reversed the Src-induced invasiveness of kidney MDCK*ts-Src* cells [22]. The local down-regulation of phosphatidylinositol-3,4,5-trisphosphate pools and downstream effector systems at the site of cell–cell contacts proved to be focal points for restraining both disruptions of junctional complexes and induction of tumor cell invasion [22]. Similarly, in the human colon cancer SW480 and HCT116 cell lines, MAGI1 overexpression stabilizes E-cadherin localization at cell–cell junctions, enhances actin stress fiber, and focal adhesion formation, increases cell adhesion to matrix proteins, and suppressed anchorage-independent growth, migration, and invasiveness [148]. Conversely, MAGI1 silencing decreases E-cadherin and β-catenin localization at cell–cell junctions, disrupts actin stress fiber and focal adhesion, and promotes anchorage-independent growth, migration, and invasion in vitro. In the breast cancer MDA-MB-231 and MCF7 cell lines, down-regulation of miR-487a restores MAGI2 expression, suppresses epithelial–mesenchymal transition (EMT), and inhibits the invasive phenotype [139]. Similarly, restitution of MAGI1 expression in glioblastoma cell lines decreases proliferation, migration, and invasion through AKT, MMP2, MMP9, and the E-cadherin/ N-cadherin/vimentin pathway [22,195].

In luminal breast cancer MCF7 cell line, downregulation of MAGI1 is associated with an increased accumulation of AMOTL2 (Angiomotin Like 2) and of E-cadherin, and to enhanced cell proliferation, but not cell migration or invasiveness [88]. This process seems to be related to an increased cell stiffness caused by actin cytoskeletal tension, leading to the activation of the p38 stress signaling pathway via AMOTL2 and ROCK. Interestingly, the Hippo and Wnt pathways were not affected. 

In endothelial cells, MAGI1 promotes focal adhesion maturation and reduces its turnover. This process involves increased endothelial cells adhesion to the extracellular matrix, via the transient increase in both RhoA and Rac1 activities, and reduces invasiveness and tubulogenesis in vitro, and suppresses angiogenesis in vivo [196]. 

##### Competitions between Scaffolding Molecules and Molecular Partners

The role of MAGIs in the assembly and the subcellular targeting of supramolecular complexes enables alternative regulation of cell signaling through the competition with scaffolding molecules orchestrating other signalosomes. In this context, MAGI3 competes with NHERF2 for the binding to the carboxyl-terminal PDZ binding motifs of both lysophosphatidic acid receptor 2 (LPAR2) and phospholipase C β-3 (PLC-β3) (Figure 2 and Figure 3, Table 1). In the HCT-116 colon cancer cell line, LPA triggers invasiveness through NHERF2 interaction with Gαq, and the activation of PLC-β3 and the downstream effectors nuclear factor-𝜅B (NF𝜅B) and c-Jun N-terminal kinase. By contrast, MAGI3 attenuated PLC-β activity and inhibited the invasive phenotype via coupling LPAR2 to both Gαq and Gα12, and potentiated LPA-induced RhoA activation [75]. The physiopathological relevance of this regulation is highlighted by the overexpression of NHERF2 and the downregulation of MAGI3 in sporadic human colorectal cancers and intestinal tumors from APC^Min/+^ mice. This antagonistic activity of NHERF2 and MAGI3 in activating PLC-β3 was also evidenced for the P2Y1 purinergic receptor [75].

On the other hand, MAGI molecular partners might compete for binding to the same MAGI interaction modules. In this connection, we have demonstrated that TRIP6—a LIM protein related to Zyxin, LPP, Ajuba, and LIMD1—competes with β-catenin for interacting with the PDZ5 of MAGI1B, leading to junctional complexes destabilization and invasiveness (Figure 2) [26]. This release of β-catenins from junctional complexes has been also associated with their nuclear translocation and the transactivation of Wnt target genes [92]. The implication of TRIP6 in tumor progression is illustrated by its overexpression in various cancers, including colorectal, hepatocellular, gastric, breast, and cervical cancers [26,92,197,198,199,200]. Besides junctional complex destabilization, we demonstrated that PI3-kinase/AKT and NF𝜅B pathways were implicated in TRIP6-induced invasiveness [26]. TRIP6 also interacts with the Hippo/YAP1 pathway. Under high mechanical tension (low cell density), TRIP6 is recruited to adherens junctions of the mammary MCF10A cells by vinculin, and competes with MOB1 for its interaction with LATS1/2, leading to YAP1 activation and cell proliferation [201]. Likewise, in cervical cancer cell lines, TRIP6 promotes proliferation and invasiveness through the YAP1 activation [202]. Whether defective MAGI/ PTEN or MAGI/ β-catenin axes are involved in these processes remains an interesting point to investigate. 

Different levels of MAGI tumor suppressor activities are displayed in Figure 3.

## 3. MAGI ceRNAs in Carcinogenesis

Interestingly, it was recently evidenced that *MAGI* gene loci are involved in the control of carcinogenesis, independently of the transcription of the corresponding MAGI scaffolding molecules, their splice variants, and alternative polyA truncated isoforms, but through transcripts that act as competing endogenous (ceRNA) RNAs. CeRNA appears as a novel regulatory mechanism between non-coding miRNA and coding RNA. These long non-coding RNAs (lncRNAs) can function as miRNA sponges, thus inhibiting the degradation of miRNAs target transcripts.

**Figure 4:**
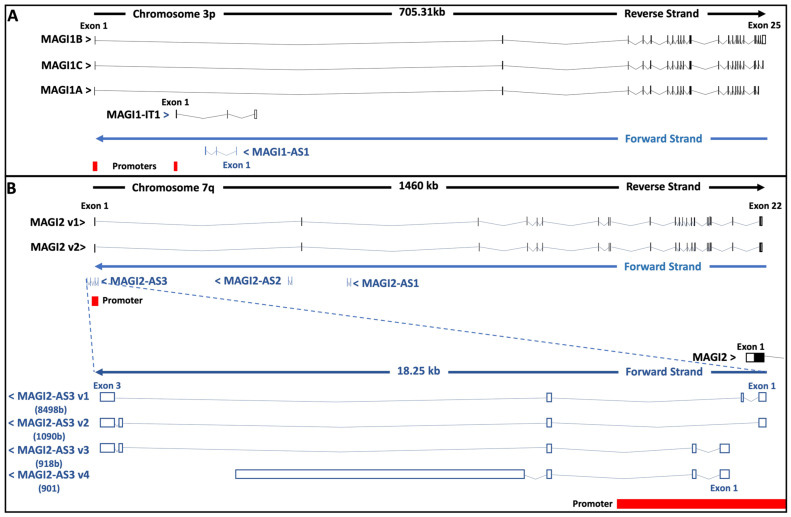
Structure of *MAGI1* (**A**) and *MAGI2* (**B**) genetic loci. (**A**) *MAGI1* maps to chromosome 3p14.1 at chr3:65,353,525-66,038,918 (GRCh38/hg38) and encompasses 25 exons. The genomic region coding for MAGI1-IT1 and MAGI1-AS1 lncRNAs are located in the sequence corresponding to *MAGI1* intron 1. (**B**) *MAGI2* gene is located on chromosome 7q21.11 at chr7:78,017,055-79,453,667 (GRCh38/hg38) and encompasses 22 exons. The genomic sequences encoding MAGI2-AS1 and MAGI2-AS2 are located in intronic regions of *MAGI2*, whereas the MAGI2-AS3 sequences encompass *MAGI2* exon 1 and/or upstream sequences. Other MAGI2-AS3 variants originating from alternative translation initiation sites or alternative splicing are not represented. The coding sequences are illustrated by filled boxes, the noncoding regions by unfilled boxes, and the intronic region by slopped thin lines. Adapted from www.ncbi.nlm.nih.gov/gene, accessed on 22 July 2021 (9223; 151877; 9863; 100505881) and www.ensembl.org, accessed on 22 July 2021 (ENSG00000151276; ENSG00000272610; NSG00000187391, ENSG00000234456).

On the search of lncRNAs involved in cardiac hypertrophy, Song et al. identified MAGI1-IT1, an intronic transcript 1 of *MAGI1* gene [203] (Figure 4). MAGI1-IT1 acts as a negative modulator of cardiac hypertrophy by sponging miR-302e which exhausts DKK1 -an antagonist of LRP6 co-receptor-, leading to inhibition of Wnt/ β-catenin signaling [204].

In non-small cell lung cancer, the accumulation of this 2585 nucleotides lncRNA is significantly correlated with tumor size, metastatic disease, shorter disease-free survival, and shorter overall survival [205]. Downregulation of MAGI1-IT1 decreases the proliferation of the human lung adenocarcinoma A549 and PC-9 cell lines. Mechanistically, MAGI1-IT1 upregulates AKT by competing with miR-512-3p [205] (Table 4). 

Similarly, MAGI1-IT1 is overexpressed in omental and mesenteric metastases of epithelial ovarian cancers as compared to primary tumors or control tissue and is positively associated with the III-IV FIGO stage. The overexpression or the downregulation of MAGI1-IT1 did not affect the proliferation of the SKOV and ES-2 ovarian cancer cell lines, in vitro and subcutaneously xenografted in nude mice. In contrast, MAGI1-IT1 promoted SKOV and ES-2 cell migration and invasion in vitro and intraperitoneal dissemination after orthotopic xenograft in nude mice [206]. MAGI1-IT1 promotes EMT, cell migration, and invasion by competitively binding miR-200a leading to the upregulation of the transcription repressors ZEB1 and ZEB2 [206] (Table 4).

MAGI1-IT1 is also overexpressed in gastric cancers and associated with a poorer patient overall survival. Mechanistically, MAGI1-IT1 sequesters miR-302d-3p enabling accumulation of its target insulin-like growth factor 1 (IGF1). Knock-down of the lncRNA in the human gastric cancer AGS and MGC-803 cell lines decreases proliferation in vitro and tumor growth in nude mice [207].

A MAGI1 antisense RNA has been identified, but to our knowledge, no physiological role has been described (Figure 4).

Three MAGI2 antisense RNAs have been identified (Figure 4). If no function has been so far attributed to MAGI2-AS1 and MAGI2-AS2, the lncRNA MAGI2-AS3 seems to act as a tumor suppressor in a tissue-dependent manner through different molecular processes, including sponging some sets of miRNAs and regulating epigenetic mechanisms (Table 4). 

In non-small cell lung carcinomas (NSCLCs), MAGI2-AS3 is downregulated and associated with tumor size, TNM stage, distant metastasis, and shorter overall survival [210,211,214,230]. In the human NSCLC H1993 cell line, the inhibition of migration and invasiveness by MAGI2-AS3 has been attributed to quenching miR-25 and upregulation of RECK (reversion inducing cysteine-rich protein with kazal motifs). Reck is an extracellular protein that may serve as a negative regulator for matrix metalloproteinase-9 [212]. Concurrently, MAGI2-AS3 may control NSCLC cell proliferation via miR-155/ SOCS-1 axis [214]. In lung squamous carcinoma cell lines, the subtype of NSCLC, MAGI2-AS3 overexpression inhibits proliferation and migration in vitro, as well as tumor growth and experimental metastasis of human adenocarcinoma A549 cells inoculated in nude mice. In this study, MAGI2-AS3 proved to sponge miR-374a/b-5p and miR-23a-3p that deplete *cell adhesion molecule 2 (CADM2)* and *PTEN* transcripts, leading to upregulation of CADM2, PTEN, caspase 3, and Bax, and reduced level of Bcl-2 [211]. MAGI2-AS3 was also reported to suppress the proliferative and invasive abilities of the A549 and PC9 NSCLC cell lines by targeting miRNA-23a-3p leading to restored expression of the PTEN tumor suppressor [210]. Interestingly, the level of MAGI2-AS3 is decreased in tumor-educated blood platelets and plasma of patients with NSCLC and is correlated with TNM stage, lymph node metastasis, and distant metastasis, making this lncRNA a potential biomarker for the noninvasive diagnosis of lung cancer [230].

In breast cancer, MAGI2-AS3 is downregulated compared to adjacent control tissues, and this accumulation is negatively correlated with histological grade, TNM stage, ER expression, PR expression, and HER-2 expression [223]. Among the clinical group of breast cancer, MAGI2-AS3 is poorly expressed in tumors with lymph node involvement [231]. Furthermore, an elevated level of MAGI2-AS3 is associated with better relapse-free survival for patients with triple-negative breast cancer subtype (absence of estrogen and progesterone receptors, no amplification of human epidermal growth factor receptor HER2) associated with a poor prognosis [232]. Mechanistically, Yang et al. showed that overexpression of the lncRNA in MDA-MB-231 and MCF-7 breast cancer cells inhibited proliferation and promoted cell apoptosis, at least partly through increased expression of Fas and Fas ligand (FasL) [223]. On the other hand, Du et al. reported that MAGI2-AS3 impedes miR-374a that exhausts the transcripts of the tumor suppressor PTEN [216].

Interestingly, in hepatocellular carcinoma (HCC), MAGI2-AS3 targets miR-374b-5p, and inhibits the proliferation and migration of HepG2, Hep3B, and MHCC-97H HCC cell lines in vitro, and the growth of HepG2 xenografted in nude mice [217]. In this study, Yin et al. identified genitalia family member 1 (SMG1) -involved in nonsense-mediated mRNA decay- as positively regulated by MAGI2-AS3, and showed that *SMG1* knockdown reverses the suppressive function of MAGI2-AS3 in HCC cells [217]. Whether, PTEN, a target of miR-374b-5p [233] is also downregulated in HCC remains an intriguing point to investigate. The ectopic expression of MAGI2-AS3 also induces the downmodulation of ROCK2 (Rho-associated coiled-coil containing protein kinase 2) at both RNA and protein levels. Although the underlying mechanisms have not been investigated, this downmodulation decreases the migration and invasiveness and induces apoptosis in the Hep3B and MHCC97-H hepatocarcinoma cell lines [224].

As observed in NSCLC and breast cancer, downregulation of MAGI2-AS3 in HCC is strongly related to tumor size, lymph node metastasis, TNM stage, and shorter overall survival [217,221]. Furthermore, plasma levels of MAGI2-AS3 are significantly lower in patients with hepatocarcinoma compared with healthy individuals and decrease in case of distant recurrence after tumor resection [224]. Thus, plasma levels of this lncRNA might improve the diagnosis and follow-up of HCC.

Likewise, MAGI2-AS3 proved also to be downregulated in gliomas and associated with shorter overall survival [234]. 

An in sillico analysis of expression profiles of lncRNA, miRNA, and mRNA, along with the clinical information of bladder carcinoma patients allowed us to identify MAGI2-AS3 as a key node encompassing 17 miRNA nodes and 46 mRNA nodes. Among the putative miRNA targets, miR-200 and miR-143 are considered tumor suppressors in human bladder cancer [226]. Nevertheless, several studies have demonstrated in this cancer that MAGI2-AS3 was downregulated and negatively associated with tumor stage [208,213,235]. In this regard, Wang et al. showed that low levels of MAGI2-AS3 were associated with a poor prognosis and that ectopic expression of MAGI2-AS3 inhibited proliferation, migration, and invasion of the human bladder cancer cells T24 and RT4 in vitro, and suppressed the growth of T24 cells in nude mice [208]. MAGI2-AS3 could serve as a competing ceRNA for miR-15b-5p that targets CCDC19 (CFAP45, cilia, and flagella associated protein 45), a tumor suppressor in bladder cancer. Rescue assays demonstrated that knockdown of *CCDC19* restored the proliferation, migration, and invasion of bladder cancer cells suppressed by MAGI2-AS3 overexpression [208]. Tang et al. also demonstrated that MAGI2-AS3 upregulates tensin 1 and inhibits the migration and invasion properties of bladder cancer cell lines by quenching miR-31-5p [213].

In the context of epithelial ovarian cancer, specifically high-grade serous ovarian carcinoma, MAGI2-AS3 acts as a tumor suppressor by sponging miR-15b-5p, miR-374a-5p, and miR-374b-5p, and may regulate the expression of their target mRNAs *PTEN*, *RECK*, *Metastasis suppressor protein 1 (MTSS1)*, *Homeobox -A5 (HOXA5)* [209]. MAGI2-AS3 might also inhibit the proliferation and invasiveness of ovarian cancer cells through downregulation of c-Myc signaling. MAGI2-AS3 is poorly expressed in ovarian cancer tissues compared with adjacent control tissue. Restoration of MAGI2-AS3 expression in the human ovarian cancer SUN8 and Caov3 cell lines depletes miR-525-5p which exhausts MXD1 (MAX dimerization protein 1). MDX1 antagonizes c-Myc-mediated transcriptional activity by competing for the binding partner MAX and by recruiting repressor complexes containing histone deacetylases [218].

A polymorphism in MAGI2-AS3 (rs7783388 GG genotype) seems to enhance colorectal cancer risk as a result of decreased binding affinity of glucocorticoid receptor to the *MAGI2-AS3* promoter, leading to lower MAGI2-AS3 accumulation [236]. In this connection, functional experiments showed that MAGI2-AS3 overexpression suppressed proliferation (cell cycle arrest at G0/G1 phase), decreased migration and invasiveness, and promoted apoptosis of the human colon cancer SW480 and SW620 cell lines [236]. In this cancer, a MAGI2-AS3 LncRNA-miRNA-mRNA network of differentially expressed genes has been recently proposed [237].

Another mechanism for the tumor suppressor activity of MAGI2-AS3 independent of miRNA sponging has also been evidenced in HCC cells [221]. MAGI2-AS3 binds and recruits the lysine demethylase 1A (KDM1A) to the *RACGAP1* promoter in an RNA-DNA manner. The demethylation of histone H3 at lysine 4 (H3K4me2) enriched in the *RACGAP1* promoter region downmodulates RacGAP, inhibits proliferation, and promotes apoptosis of HEPG2 cells in vitro, and decreases tumor growth in nude mice [221]. Likewise, MAGI2-AS3 impairs leukemia stem cells’ self-renewal through recruitment of TET2 (Tet methylcytosine dioxygenase 2, involved in cytosine demethylation) to the *LGIR1* promoter. The enhanced expression of LRIG1 (leucine-rich repeats and immunoglobulin-like domains 1) might negatively regulate the EGFR, MET, and RET receptor tyrosine kinases [222]. The low expression of MAGI2-AS3 in these malignant cells favors the self-renewal and the maintenance of the clonal hierarchy of acute leukemia stem cells. In esophageal cancer, MAGI2-AS3 can down-regulate HOXB7 expression by recruiting the histone-lysine N-methyltransferase EZH2 (enhancer of zeste homolog 2) to promote the tri-methylation of lysine 27 on histone H3 in the *HOXB7* promoter region. The ensuing decreased transcription of *HOXB7* inhibits proliferation and radio-resistance of esophageal cancer cell lines in vitro and in vivo [219]. Both MAGI2-AS3 and EZH2 are expressed at low levels in esophageal carcinomas. In clear cell renal cell carcinoma, the downregulation of MAGI2-AS3 is associated with a poor prognosis. MAGI2-AS3 forms a nucleoprotein complex with HEY1 (Hes related family BHLH transcription factor with YRPW motif 1) in the nucleus and impairs its transcriptional repressor activity on *ACY1* (aminoacylase 1) promoter region [220]. Restoration of ACY1 expression by MAGI2-AS3 decreases viability, expression of EMT markers, migration, and vasculogenic mimicry in vitro, and reduces the growth and angiogenesis of the human clear cell renal cell carcinoma RLC-310 cell line xenografted in nude mice [220].

Interestingly in breast cancer cells, MAGI2-AS3 proves to act as a cis-acting regulatory element, decreasing *MAGI2* promoter methylation through TET1 recruitment. Restoration of MAGI2-AS3 expression in the MCF-7 mammary cell line induces MAGI2 expression, leading to the inactivation of AKT and Wnt/β-catenin pathways and impaired cell proliferation and migration [128].

All these studies report the downregulation of MAGI2-AS3 during carcinogenesis and support a role of this lncRNA as a tumor suppressor in adrenal, brain, breast, bladder, liver, lung, colorectal, prostate, pancreatic, ovarian, and renal cancers [208,209,210,213,214,217,220,222,223,230,232,234,235,236,237,238,239].

In contrast, MAGI2-AS3 proved to be overexpressed in gastric cancers and nasopharyngeal carcinomas [225,228,240]. MAGI2-AS3 overexpression enhances nasopharyngeal cells proliferation and migration, and their resistance to cisplatin. This process is related to miR-218-5p downregulation, leading to increased accumulation of GDPD5 (glycerophosphodiester phosphodiesterase domain containing 5) that cleaves the glycosylphosphatidylinositol anchoring proteins to the plasma membrane, and SEC61A1 (SEC61 translocon subunit α1) involved in the insertion of polypeptides into the endoplasmic reticulum [228]. In gastric cancer, MAGI2-AS3 is negatively associated with overall survival and disease-free survival of cancer patients and promotes epithelial–mesenchymal transition (EMT), cell migration, and invasiveness by sponging miR-141/200a and upregulating the ZEB1 transcription factor [225]. In the metastatic lymph node from a gastric cancer HGC-27 cell line, MAGI2-AS3 overexpression might be caused by the transcriptional regulator bromodomain containing 4 (BRD4) and its interaction with histone H3 acetylated at lysine 27, enriched in the promoter region *MAGI2-AS3* [225]. In the HCT116 and RKO human colon cancer cells, depletion of MAGI2-AS3 restrains proliferation and increases apoptosis [229]. This effect seems linked to the quenching of miR-3163 and the upregulation of TMEM106B.

In cervical cancer, conflicting results have been reported. Wang et al. showed that the expression of lncRNA MAGI2-AS3 was reduced in tumors compared to control tissues and that the promoter region of the lncRNA was hypermethylated [241]. In this study, the prognostic value of MAGI2-AS3 was not statistically significant. Similarly, Hou et al. evidenced a decreased accumulation of MAGI2-AS3 in cervix squamous cell carcinomas compared with control tissue [215]. They further demonstrated that MAGI2-AS3 inhibited SiHa and HeLa cell invasion and migration by sponging miR-223 leading to up-regulation of the tumor suppressor EPB41L3. In contrast, Liu et al. noticed that high levels of MAGI2-AS3 were associated with the poor survival of patients with cervical squamous cell carcinoma [242]. In the human cervical cancer C-33A cells, MAGI2-AS3 overexpression promotes cell proliferation by affecting the cell cycle through CDK6 upregulation [242]. 

These dual effects of MAGI2-AS3 as a tumor promoter or suppressor might be related to the cellular context, including tissue origin, the patterns of mRNA/ miRNA expression, the genetic defects, the pathways dysregulated but also the existence of different variants of this lncRNA, -due to different translation initiation sites, and alternative splicing- that might target distinct miRNAs. It appears therefore of major importance to perform the fine analysis of the pattern of expression of these variants in relation to physiopathology.

## 4. Conclusions, Perspectives

**Figure 5:**
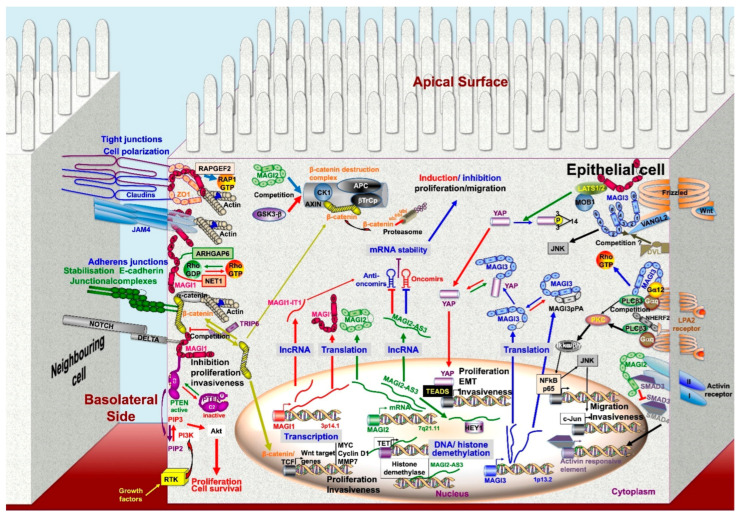
Schematic overview of MAGI signaling dysregulation in cancer, at the levels of MAGIs themselves (transcription, RNA maturation, post-translational modifications), of their interacting partners and effectors systems including signalization, gene expression and epigenetics, and the biological impact of these dysregulations. For details see the text.

Scaffolding molecules exert a critical role in orchestrating cellular response by increasing the efficiency and selectivity of signal transduction. The emerging role of PDZ domain molecules in carcinogenesis might be related to some redundancy between these different protein families. In this concern, PTEN does interact with the three MAGIs, but also with other scaffolding molecules such as DLG1 and SCRIB. This further highlights the essential role of these scaffolds in the control of signal transduction. Nevertheless, we report here strong evidence concerning the downmodulation of MAGIs in various types of cancers and their physiopathological implications (Figure 3 and Figure 5). Biochemical, biophysical, and mathematical modeling approaches have allowed one to identify molecular partners and effectors systems driven by scaffolding molecules, mainly through a global determinist approach. A challenge in our knowledge of these networks concerns the characterization of the dynamic of these signalosomes and their composition at the molecular level in their native environment. Molecular noise, stochasticity in the assembly of these multiprotein complexes could contribute to generating cellular signaling diversity. For instance, interdomain allostery, where binding to one PDZ domain changes the affinity at other domains, should modify the selectivity for other ligands. Using an experimental approach mimicking the anchoring of scaffold protein to the plasma membrane, Erlendsson et al. evidenced two-three higher orders of magnitude in intrinsic binding affinity than isolated PDZ interaction [243]. The limited amount of each molecular partner, their affinity, their diversity, their bioavailability in terms of subcellular localization or commitment in other scaffolds, their posttranslational modifications might contribute in a part of randomness in the assembly of these complexes. McCam et al. reported that the scaffolding protein PSD-95 does not stabilize specific complexes, but rather increases the frequency of short-lived binding events [244]. Thus, these complexes might only infrequently contain all the components necessary for signal transmission. Furthermore, these interactions seem to occur under non-equilibrium conditions [243]. Whether conceptualization of protein scaffolds as molecular machine and cell signaling as engineering wiring proved effective, these generic snapshots do not take into account the cellular context, either that real life is in multiple dimensions. In an editorial, Frank Gannon made a parallel between biology and physics before the advent of quantum physics [245]. A change in paradigm in biology might concern the dynamic integration of these molecular interactions, the self-organizing nature of the assembly, the abundance of partners and competitors, and the plasticity of these components using more probabilistic and stochastic approaches [246]. Much work remains to integrate all these different aspects to understand this networking leading to cellular outcomes and heterogeneity under physiological and pathological conditions. Nevertheless, targeting the multiple levels of regulation of MAGI scaffolding molecules enables unique opportunities to modulate simultaneously several pro/suppressive oncogenic signaling pathways controlling carcinogenesis (Figure 5). Hypermethylation of CpG islands in promoters is a common feature of neoplastic progression. In this context, a series of DNA demethylating agents such as 5-azacitidine, 5-aza-2-deoxycytidine (decitabine), 4′-thio-2′-deoxycytidine (TdCyd), and hydralazine are under clinical trials (https://clinicaltrials.gov, accessed on 6 July 2021). Such compounds might reactivate MAGI expression silenced by promoter methylation. The recent success of the RNA vaccine demonstrates the technological feasibility of RNA delivery in humans that could be applied to selectively exhaust some oncomiRs which sponge transcripts encoding tumor suppressors, including *MAGIs*. Decreased methylation in *MAGI3* large exon is correlated with the accumulation of the MAGI3pPA pro-oncogenic variant in breast cancers. Thus, targeting RNA m6A demethylases, such as ALKBH5 which is overexpressed in breast cancer and promotes stem cell maintenance [247,248,249,250] might restore MAGI3 full-length expression, hampering YAP1 signaling and promoting the PTEN tumor suppressor pathway. In this context, some inhibitors of ALKBH5 are under development and proved to efficiently exert antiproliferative activity in a cancer-cell-type-selective manner [251]. As far as MAGI1 is wild-type, its accumulation could be raised by Cox2 inhibitors leading to increased PTEN stability and activity. In this connection, the regulation of endothelial cell activity, and the tuning of vascular permeability and angiogenesis by MAGI1 might constitute another level to control carcinogenesis [166]. Growing and promising efforts are devoted to evaluating the druggability of PDZ domains [7,8]. Such an approach could benefit neurological, viral, and oncological disorders. In this connection, using a structure-based computational design framework that models peptide flexibility, Zheng et al. designed peptides that bind the PDZ2 of NHERF2 but do not interact with the PDZ5 of MAGI3 [252]. These peptides might selectively hamper NHERF2/ LPA2R interaction and oncogenic signaling, without affecting LPA2R/ MAGI3 biological activity. Alternatively, some ligands of PDZ domains could be neutralized by cell-penetrable nanobodies (transbodies) favoring selective interaction of competing molecules, e.g., TRIP6 vs β-catenin. These approaches open up new perspectives in precision medicine to assess individual tumor responses to these targeted therapeutic agents. Interestingly, besides encoding scaffolding molecules, *MAGI1* and *MAGI2* genomic loci also generate lncRNAs that modulate neoplastic progression and might serve as non-invasive biomarkers of cancer progression [224,230]. These observations clearly illustrate that, as revealed by the human genome sequencing, the complexity of an organism is not solely a matter of intrinsic gene number, but rather reflects the functional role of epigenetic processes, non-coding genetic sequences, RNA editing, alternative splice, posttranslational modifications, multiprotein complexes assembly, and dynamic self-organizing systems that go well beyond the set of instructions of the “genetic program”.

## Figures and Tables

**Table 1:** A partial list of MAGI-interacting partners, domains involved, and functional consequences.

Molecular Partners	Full Name	Function	MAGI1	Interaction MAGI2	MAGI3	MAGI Binding Domain *	Biological Impact	References
**Receptor Ligands, Membrane Receptors, Cell Adhesion Molecules**								
*ADAM17; TACE*	Disintegrin and metalloproteinase domain-containing protein 17; ADAM17,	Membrane-anchored metalloproteinase	+	ND	ND	PDZ2	Involved (with ɣ-secretase) in coxsackie virus and adenovirus receptor (CAR Ex8)	[48]
*ADGRB1; BAI1*	Adhesion G protein-coupled receptor B1; Brain-specific angiogenesis inhibitor-1	G-protein-coupled receptor involved in inflammation, tumorigenesis, and phagocytosis. Mobilizes Rho signaling pathway through Gα12/13 and Rac1 pathways via ELMO/DOCK GEF.	+	+	+	PDZ4	BAI1 PDZ-binding motif required for Rho signaling, association with β-Arrestin2, and receptor ubiquitination. MAGI3 potentiates ERK signaling by a constitutively active BAI1. MAGI1 was originally designed BAI1-associated protein 1	[43,49]
*ADRB1*	Beta-1 adrenergic receptor (β1AR); Adrenoceptor beta 1;	G protein-coupled receptor (GPCR), a receptor for epinephrine and norepinephrine	+	+	+	PDZ1 (MAGI1, 3); PDZ1 and PDZ2 (MAGI2)	Interaction MAGI2/ β1AR enhanced by agonist stimulation. MAGI2 increases agonist-induced β1AR internalization. Ternary complex involves MAGI2/ β1AR/ β-catenin. MAGI3 impairs β1AR-mediated ERK1/2 activation but has no effect on cAMP generation or β1AR internalization	[50,51]
*ADRB2*	Beta-2 adrenergic receptor (β2AR)	G protein-coupled receptor (GPCR)	ND	ND	+	PDZ5	Agonist stimulation of β2AR promotes MAGI3/ β2AR interaction (dependent on β2AR phosphorylation by GRK). MAGI3 inhibits β2AR-mediated ERK activation.	[52]
*ActRIIA*	Activin A receptor type 2A	Ser/Thr kinase, a component of the heterodimeric receptor for activin, a member of the TGFβ family	ND	+	ND	PDZ5	MAGI2 interacts with activin type 2 receptor via its PDZ5 and with SMAD3 via its WW domains. MAGI2 negatively regulates activin/ SMAD3 -induced gene transcription	[53]
*CACNG2; Stargazin*	Voltage-dependent calcium channel gamma-2 subunit; Stargazin	Type I transmembrane AMPA receptor regulatory protein. Promotes targeting of AMPA-selective glutamate receptors; Modulates their gating properties.	ND	+	+	PDZ1, PDZ3, and PDZ5	Stargazin recruits MAGI2 to cell membranes and cell–cell contact. MAGI2 might act as a physical link between AMPAR/ AMPAR regulators complexes and synaptic adherens junctions	[54]
*CTNNB1*	Catenin beta 1; Beta-catenin	Component of the protein complex that constitutes adherens junctions, involved in cadherin-mediated cell–cell contact and tissue integrity. Effector system of the canonical Wnt signaling pathway: coactivator for the TCF/LEF family of transcription factors and Wnt target genes (Cyclin D1, c-Myc, MMP7).	+	+	+	PDZ5	MAGIs are recruited to cadherin junctional complexes by PDZ5 binding to β-catenin. The interaction of PTEN with MAGI PDZ2 allows the tuning of PtdIns(3,4,5)P3 and downstream effectors with PH domain (e.g., AKT, RhoGEF) stabilizing junctional complexes and inhibiting invasiveness. MAGIs also restrain β-catenin nuclear translocation and Wnt target genes transactivation	[20,21,22,35,36,37,55,56]
*CTNND2; NPRAP*	Catenin delta 2; Delta-catenin	Member of the armadillo protein family, involved in cell–cell contacts and signal transduction	ND	+	ND	PDZ5	Junctional organization	[57]
*CDH23*	Cadherin-23	Member of the cadherin superfamily; Involved in the organization of the stereocilia bundle of hair cells in the cochlea	+	−	−	PDZ4	MAGI1, PIST, and harmonin collaborate in intracellular trafficking and plasma membrane targeting of cadherin 23	[58,59]
*CRHR1; CRFR1*	Corticotropin-releasing hormone receptor 1;	G-protein (Gα) coupled receptor, binds neuropeptides of the corticotropin-releasing hormone family	+	+	+	PDZ1	Receptor clustering; MAGIs regulate CRFR1 internalization by mediating β-arrestin recruitment. MAGI modulate CRFR1 signaling via the MAPK pathway but not cAMP formation	[60,61,62]
*CXADR; CAR*	Coxsackievirus and adenovirus receptor	Component of epithelial apical junction complexes, a membrane receptor for group B coxsackieviruses, and subgroup C adenoviruses. Isoforms originate from alternative splicing, e.g., CAR Ex8	+	ND	ND	PDZ1 and PDZ3	CAR Ex8 localizes to the apical surface of epithelial cells. Interaction with MAGI1 PDZ1 protects CAR Ex8 from degradation, whereas MAGI PDZ3 interaction triggers CAR Ex8 cleavage by ADAM17.	[48]
*DLL1*	Delta-like protein; Delta1	Involved in cell–cell communication. Delta proteins activate Notch through their extracellular domains; the intracellular domains also exert biological functions.	+	+	+	PDZ4	MAGI1 recruits Delta1 to adherens junctions through its interaction with β-catenin. In Zebrafish, MAGI1, 2, and 3 interact with Delta1 and control neuron migration	[63,64,65]
*ERBB4; HER4*	Receptor tyrosine-protein kinase erbB-4	Member of EGF receptor family tyrosine kinases. Binds to and is activated by neuregulins. Mutated in various cancer types	+	+	ND	PDZ1	MAGI1, 2 bind ERBB4 and R-PTP-zeta, and form a ternary phosphotyrosine kinase/ phosphotyrosine phosphatase complex. Bring ERBB4 and R-PTP-zeta to specific cellular domains. ERBB4 phosphorylation of MAGI enhanced by ERB4 ligand.	[66]
*FZD4*	Frizzled 4, Fz-4	Receptor for Wnt proteins; Activates disheveled proteins leading to nuclear accumulation of β-catenin and transactivation of Wnt target genes	+	ND	+	PDZ1	MAGI3 forms a ternary complex with Fz-4 and VANGL2 (involved in planar cell polarity) at cell–cell contact. Both Fz-4 and Vangl2 interact with MAGI3 PDZ1suggesting MAGI3 oligomerization. This ternary complex activates JNK via Rac1	[67,68,69]
*GRIN2A; NMDAR2A; NR2A*	Glutamate receptor ionotropic, NMDA 2A	Component of NMDA receptor complexes, ligand-gated ion channels with high calcium permeability, and voltage-dependent sensitivity to magnesium.	ND	+	ND	PDZ5	Clustering of NMDA receptors? Required for NMDA receptor-induced RhoA. Hampered NMDA 2 internalization.	[30,70,71]
*HTR2A*	5-Hydroxytryptamine receptor 2A	One of the receptors for serotonin (this also binds mescaline and LSD psychoactive substances); Gq/G11-protein-coupled, increases cellular levels of IP3 and DAG via PLC and modulates PKC activity and Ca2+ release from intracellular stores	+	+	+	PDZ nd	MAGIs differentially regulate 5-HT2AR activity: MAGI1, 3 favor receptor internalization; MAGI2, 3 enhance receptor trafficking. MAGIs increase PLC-β3 recruitment to the receptor and IP3 formation. MAGI knockdown increases ERK1/2 activation by 5-HT-2A, independently of the PDZ binding motif	[72]
*IGSF5; JAM4*	Immunoglobulin superfamily member 5; Junctional adhesion molecule 4	Cell adhesion molecule at tight junctions of kidney glomerulus and small intestinal epithelial cells. Mediates calcium-independent homophilic adhesion.	+	ND	ND	PDZ1 and PDZ4	MAGI1 induces clustering of IgSF5 and strengthens cell adhesion. May regulate the permeability of kidney glomerulus and small intestinal epithelium	[73]
*LRP2; Megalin*	Low-density lipoprotein receptor-related protein 2; Megalin	Endocytic receptor expressed in absorptive epithelial tissues. Involved in reuptake of sterols, lipoproteins, vitamin-binding proteins, and hormones	+	ND	ND	PDZ5		[23]
*LPAR2; LPA2 receptor*	Lysophosphatidic acid receptor 2	G protein (Gi/Go, G12/G13, & Gq)-coupled receptor. Involved in phospholipase C-beta (PLC-β) signaling pathway	−	+	+	PDZ5	MAGI3 inhibits NHERF2 –induced migration and invasion of colon cancer cells by competing with NHERF2 for binding to LPAR2 and its downstream effector PLC-β3, leading to decreased NFkB and JNK activities	[74,75]
*NGFR; TNFRSF16; P75NTR*	Tumor necrosis factor receptor superfamily member 16; NGF receptor	Low-affinity receptor for neurotrophins (NGF, BDNF, NTF3, and NTF4). Mediates RhoA-dependent inhibition of growth of regenerating axons.	+	−	−	PDZ0	MAGI1 interacts with NGF-R and SHC, positive regulation of NGF-stimulated SHC-ERK pathway	[28]
*NLGN1*	Neuroligin-1	Neuronal cell surface protein. Transsynaptic adhesion molecules. Role in synapse function and synaptic signal transmission.	+	+	+	PDZ1	S-SCAM (MAGI2) is tethered by β-catenin and triggers the accumulation of neuroligin, which subsequently recruits PSD-95 to synapses	[30,76,77]
*NPHS1; Nephrin*	Nephrin; Renal glomerulus-specific cell adhesion receptor	Transmembrane protein, member of the immunoglobulin family of cell adhesion molecules. Involved in glomerular filtration barrier in the kidney (slit diaphragm of glomerular podocytes)	+	+	-	PDZ3 (MAGI1); Indirect or PDZ3-5 (MAGI2)	In podocytes, MAGI1 connects Nephrin and JAM4 to the actin cytoskeleton, critical for stable assembly of the slit diaphragm. Nephrin/ MAGI1 interaction enhances Rap1 activation, essential for maintaining slit diaphragm function.	[78,79,80,81,82]
*PTPRZ1; RPTPB; RPTPbeta*	Receptor-type tyrosine-protein phosphatase zeta; R-PTP-zeta	Role in cell adhesion, neurite growth, and neuronal differentiation, blood vessel remodeling, and angiogenesis	+	+	+	PDZ2	Favors interactions of R-PTP-zeta with its substrates at the plasma membrane. MAGI1, 2 form a ternary phosphotyrosine kinase/ phosphotyrosine phosphatase complex with ERBB4 and R-PTP-zeta. R-PTP-zeta dephosphorylates Tyr-373 and Tyr-858 of MAGI1	[66,83,84]
*SDK1; Sidekick-1*	Protein sidekick-1	Transmembrane protein, immunoglobulin family of cell adhesion molecules; promotes synaptic connectivity via homophilic interactions	+	+	ND	WW and PDZ4 and/or PDZ5; PDZ2 and/or PDZ3	Localizes Sidekick1, 2 in distinct subsets of retinal synapses. Upregulation of Sidekick during focal segmental glomerulosclerosis impairs MAGI1 function, promotes actin cytoskeleton disruption, leading to podocyte dysfunction	[27,85]
*TGFA; TGF-alpha*	Protransforming growth factor alpha	Growth factor; ligand for EGFR; acts as transmembrane-bound or a soluble ligand	−	+	+	PDZ1	Role in the trafficking of TGF-alpha to the cell surface in polarized epithelial cells	[33]
*VANGL2, LTAP*	Vang-like protein 2 (Vangl2); Loop-tail-associated protein (LTAP)	Membrane protein; Involved in regulation of planar cell polarity (non-canonical Wnt signaling pathway)	ND	+	+	PDZ1	MAGI3 forms a ternary complex with Fz-4 and Vangl2 at cell–cell contact and activates JNK via Rac1. Both Fz-4 and Vangl2 interact with MAGI3 PDZ1 suggesting MAGI3 oligomerization. In podocytes, MAGI2 forms a ternary complex with nephrin and Vangl2	[68,86]
*VIPR1; VPAC1*	Vasoactive intestinal polypeptide receptor 1; VIP-R-1	G protein-coupled receptor; receptor for vasoactive intestinal peptide, involved in smooth muscle relaxation, exocrine and endocrine secretion, and hydro-electrolytic flux	ND	+	ND	PDZ1 and/or PDZ2	MAGI2 reduces VIP-R-1 activation, leading to decreased receptor internalization (GRK dependent). Recruitment of VIP-R-1 close to CFTR enables localized cAMP generation and hydroelectrolytic secretion with a minimal cellular response.	[87]
**Adaptors, Scaffolding Molecules**								
*AMOTL2*	Angiomotin-like protein 2	Member of the angiomotin membrane-associated scaffold proteins. Involved in cadherin linkage to the cytoskeleton and in YAP1 signaling	+	ND	ND	2nd WW	MAGI1 downregulation is associated with enhanced accumulation of AMOTL2 and E-cadherin in MCF7 cells. Junctional dysfunction and cytoskeletal tension might activate the p38 stress pathway and tumorigenesis	[88]
*ARRB2; BAAR2*	Arrestin beta 2; Beta-arrestin-2	Member of arrestin protein family; scaffolding molecule; involved in agonist-mediated desensitization of GPCR, and in signaling regulation, e.g., TGFβ, MAPK, PTEN, AKT, NFkB, TP53	+	ND	ND	PDZ nd	Role in regulating β-arrestin-2 recruitment to CRFR1 and receptor signaling. Involved in receptor/β-arrestin complex stability and trafficking, and in signaling functions of receptors.	[61,62]
*Axin*	Axin	Tumor suppressor involved in the control of Wnt pathway. Cytoplasmic protein interacting with APC, β-catenin, GSK 3β, PP 2A, and itself. Component of the complexities involved in β-catenin destruction.	ND	+	ND	GuK	MAGI2 forms a complex with β-catenin and Axin and competes with GSK3β for Axin-binding. MAGI2 may protect β-catenin from degradation by inhibiting its phosphorylation by GSK3β.	[89]
*DLGAP4; SAPAP4*	Disks large-associated protein 4	Membrane-associated guanylate kinase found at the postsynaptic density in neuronal cells	ND	+	ND	GuK	Interaction of DLGAP with MAGI2 (S-SCAM)	[30]
*DLG4; SAP-90; PSD95*	Disks large homolog 4; Synapse-associated protein 90; Post-synaptic density protein 95	MAGUK family, contains 1 domain SH3, 1 Guk domain, and 3 PDZ domains. Postsynaptic scaffolding protein involved in synaptogenesis and synaptic plasticity	ND	+	ND	PDZ4 + PDZ5	The Guk domain of PSD95/ SAP90 interacts with PDZ4+PDZ5 of MAGI2. Role in clustering NMDA receptor?	[40]
*FCHSD2*	F-BAR and double SH3 domains protein 2; Carom	Adapter protein. Promotes endocytosis of EGFR and down-regulation of EGFR signaling. Contributes to internalization of integrin β1 and transferrin receptor. Binds to membranes enriched in PtdIns(3,4)-P2 or PtdIns(3,4,5)P3. Promotes actin polymerization.	+	ND	ND	PDZ5	MAGI1 colocalizes with Carom at immature cell contacts of epithelial cells. MAGI1 competes with CASK (calcium/calmodulin-dependent Ser protein kinase) for Carom binding.	[25]
*MAGI2; SSCAM; ACVRIP1; AIP1; ARIP1*	Membrane-associated guanylate kinase, WW, and PDZ domain-containing protein 2	Scaffolding molecule containing 1 Guk domain, 2 WW domains, and 6 PDZ domains	ND	+	ND	PDZ4 + PDZ5	MAGI2 dimerization might be involved in scaffolding, clustering, signaling	[30,40,44]
*MAGI3*	Membrane-associated guanylate kinase, WW, and PDZ domain-containing protein 3	Scaffolding molecule containing 1 Guk domain, 2 WW domains, and 6 PDZ domains	ND	ND	+	ND	Frizzled-4 and Vangl2 form a ternary complex with MAGI3 via binding to PDZ1, suggesting MAGI3 oligomerization. Assumption supported by the interaction of wild-type MAGI3 with MAGI3pPA	[68]
*MAGI3pPA*	MAGI3 with premature polyadenylation	A truncated form of MAGI3 (depletion PDZ2-5) resulting from the use of a cryptic intronic polyadenylation signal	ND	ND	+	ND	Prooncogenic; competition with full-length MAGI3 for controlling YAP1 signaling; increases nuclear YAP1 localization. Expressed especially in breast cancer	[42,90]
*CNKSR2; KSR2*	Connector enhancer of kinase suppressor of Ras 2	Scaffolding protein with SAM, PDZ, and PH domains. Involved in MAPK pathways	ND	+	ND	PDZ4 and/or PDZ5	Role in linking cell surface receptors to MAPK pathways?	[91]
*SHC*	SHC-transforming protein	Couple activated receptor tyrosine kinases to the RAS pathway by recruitment of GRB2/SOS complex	+	ND	ND	PDZ4 and/or PDZ5	MAGI1 interacts directly with NGF-R and SHC, positive regulation of NGF-stimulated SHC-ERK pathway	[28]
*TRIP6; ZRP1*	Thyroid receptor-interacting protein 6	Member of the zyxin family contains 3 LIM domains. Localizes to focal adhesion and actin stress fibers. Regulates lysophosphatidic acid-induced cell migration. Modulates gene expression by nuclear translocation and interaction with some transcriptional factors, e.g., NFkB	+	ND	ND	PDZ5	TRIP6 competes with β-catenin for binding MAGI1 PDZ5. Hence, TRIP6 impairs PTEN recruitment to E-cadherin/ β-catenin complexes and adherens junctions stabilization. In conjunction with AKT and NFkB activation by its core protein, TRIP6 promotes invasiveness. Displacement from MAGI1 allows β-catenin nuclear translocation and Wnt oncogenic signaling	[26,92]
**Intracellular Signaling Proteins**								
*ARHGAP6*	Rho GTPase-activating protein 6	GTPase activator for the Rho-type GTPases; Involved in the regulation of actin polymerization at the plasma membrane	+	ND	ND	PDZ2	The antagonistic activities of NET1 and ARHGAP6 and their recruitment in the same molecular scaffold via two adjacent MAGI1 PDZ domains allow the fine-tuning of Rho signaling. The four last residues of NET1 and ARHGAP6 are identical, thus their selective binding to PDZ1 and PDZ2 of MAGI1 must be defined by residues upstream.	[67]
*ATN1*	Atrophin	Transcriptional corepressor. Associated with dentatorubral pallidoluysian atrophy, a rare neurodegenerative disorder	+	+	ND	WW	The first designation of MAGI2 was atrophin-1-interacting protein 1 (AIP1) as a novel partner of atrophin	[44]
*KRAS; Ki-Ras*	GTPase KRas; K-Ras 2	Proto-oncogene member of the small GTPase superfamily; upstream effector of PI3K and MAPK signaling pathways.	+	ND	ND	PDZ1	Role in K-Ras 2 targeting to plasma membrane? Interaction not validated in mammalian cells	[29]
*LATS1/2*	Serine/threonine-protein kinase LATS1/2; Large tumor suppressor homolog 1/ 2	Ser, Thr kinases. Negative regulators of YAP1 (Hippo signaling pathway). Involved in the control of cell cycle, a negative regulator of CDK1/cyclin A	+	+	+	WW	The PY motifs of LATS1/2 interact with the WW domain tandem of MAGIs. This tandem enables high affinity and specificity towards their ligands.	[34,93]
*MAP2K1, MEK1*	Dual specificity mitogen-activated protein kinase kinase 1; MEK1 MKK1	Dual specificity protein kinase. Phosphorylates a Thr and a Tyr residue in a Thr-Glu-Tyr sequence of MAPK3/ERK1 and MAPK1/ERK2, leading to their activation and transduction of the signal within the MAPK/ERK cascade	+	+	+	WW; GuK	MEK1 is necessary for PTEN membrane recruitment as part of a ternary complex involving MAGI1. Complex formation requires MEK1 phosphorylation by ERK and leads to the negative regulation of the AKT pathway	[94]
*NET1; ARHGEF8*	Neuroepithelial cell-transforming gene 1 protein	Rho guanine nucleotide exchange factor	+	ND	ND	PDZ1	The antagonistic activities of NET1 and ARHGAP6 and their recruitment in the same scaffold via two adjacent MAGI1 PDZ domains allow the fine-tuning of Rho signaling. The four last residues of NET1 and ARHGAP6 are identical, thus their selective binding to PDZ1 and PDZ2 of MAGI1 must be defined by residues upstream.	[67,95]
*PLCB3*	1-phosphatidylinositol 4,5-bisphosphate phosphodiesterase beta-3; PLC-beta-3	Member of the phospholipase C-β family generates DAG and InsP3 from phosphatidylinositol in response to G-protein-linked receptor stimulation.	+	+	+	PDZ nd	MAGI3 competes with NHERF2 for binding to both LPAR2 and PLC-β3, decreases the activities of PLC-β3 and its downstream effector NFκB by facilitating LPAR2 coupling to different G proteins	[72,75]
*PTEN*	Phosphatase and Tensin homolog deleted on chromosome TEN	Phosphatidylinositol 3,4,5-trisphosphate 3-phosphatase and dual-specificity protein phosphatase; Tumor suppressor	+	+	+	PDZ2	PTEN stabilization, PTEN recruitment to high molecular weight molecular complexes, including E-cadherin junctional complexes. Stabilization of cell–cell junctions, decreased cell migration	[22,37,96,97,98]
*PTPN14; PTPD2; PEZ*	Protein tyrosine phosphatase non-receptor type 14	Tyr phosphatase with a FERM domain (cytoskeletal-associated proteins). Putative tumor suppressor. Dephosphorylates β-catenin at junctional complexes and stabilizes cell–cell contacts. Negatively regulates YAP1 (phosphatase dependent and independent manner)	+/−	+	+	WW	The PY motifs of PTPN14 interact with the WW domain tandem of MAGIs. This tandem enables high affinity and specificity towards their ligands. The inter-domain between the WW tandem might modulate the affinity of WW domains	[34]
*PTPN21; PTPD1*	Protein tyrosine phosphatase non-receptor Type 21	Protein tyrosine phosphatase. Contains a FERM found in cytoskeletal-associated proteins.	ND	+	+	WW	The PY motifs of PTPN21 interact with the WW domain tandem of MAGIs. This tandem enables high affinity and specificity towards their ligands.	[34]
*RAPGEF2; NRap GEP; PDZ-GEF1*	Rap guanine nucleotide exchange factor 2; RA-GEF-1	Guanine nucleotide exchange factor (GEF); activates the Rap and Ras family of small GTPases by exchanging bound GDP for free GTP in a cAMP-dependent manner	+	+	ND	PDZ0 (MAGI1; PDZ1 (MAGI2)	MAGI1 recruits RA-GEF-1 at tight junctions in epithelial cells. In endothelial cells, MAGI1 is required for Rap1 activation and adherens junction formation. Translocation of MAGI1 to cell–cell contacts is ascribed to its interaction with β-catenin. MAGI2 co-expressed with RA-GEF-1 enhances activation of Rap1 in podocytes, signaling essential for normal podocyte function	[99,100,101,102]
*SMAD3*	Mothers against decapentaplegic homolog 3; SMAD family member 3	Effector of TGFβ and activin type 1 receptor kinases. Transmits signals from the cell surface to the nucleus. Forms heterodimer with SMAD4 and activates transcription.	ND	+	ND	WW	MAGI2 interacts with activin type 2 receptor via its PDZ5 and with SMAD3 via its WW domains. MAGI2 negatively regulates activin/ SMAD3 -induced gene transcription	[53]
*YAP1*	Yes-associated protein 1	Regulator for TEAD transcription factors; coactivator and corepressor of gene expression downstream Hippo signaling pathway; Involved in cell growth, anchorage-independent growth, and EMT	ND	ND	+	PDZ5	MAGI3 interacts with and sequesters YAP1 oncoprotein in the cytoplasm, inhibiting YAP1-induced malignant transformation of human mammary epithelial cells.	[42,90]
**Actin Associated Proteins**								
*ACTN4*	Alpha-actinin-4	Actin-binding protein	+	ND	ND	PDZ5	Role in actin cytoskeleton dynamics?	[24]
*SYNPO*	Synaptopodin	Actin-associated protein, involved in actin-based cell shape and motility	+	ND	ND	2nd WW	Role in actin cytoskeleton dynamics?	[24]
**Posttranslational Modifications**								
*CASP3*	Caspase-3; CASP-3	Cysteine-aspartic acid protease. Effector caspase is involved in the execution phase of apoptosis. Activated by proteolytic cleavage. CASP-3 shares several substrates with CASP-7 but exerts distinct functions during apoptosis.	+	+	ND	Asp761 (MAGI1)	MAGI1 is cleaved by CASP-3 at Asp761, between PDZ2 and PDZ3. The N-term fragment translocates in the cytoplasm and the C-term accumulates in the nucleus. MAGI1 cleavages induce disruption of cell–cell contacts and apoptosis. MAGI2 cleavage site was not determined.	[103,104]
*CASP7*	Caspase7; CASP-7	Cysteine-aspartic acid protease. Effector caspase is involved in the execution phase of apoptosis. Activated by proteolytic cleavage. CASP-7 shares several substrates with CASP-3 but exerts distinct functions during apoptosis.	+	ND	ND	Asp761	MAGI1 is cleaved by CASP-7 at Asp761, generating 97 kDa and 68 kDa fragments. The N-term fragment translocates in the cytoplasm and the C-term accumulates in the nucleus. MAGI1 cleavages induce disruption of cell–cell contacts and apoptosis.	[103]
*RPS6KA1, P90RSK*	Ribosomal protein S6 kinase alpha-1; S6K-alpha-1	Ser/Thr kinase. Acts downstream ERK1/2. Mediates mitogenic and stress-induced activation via CREB1. Regulates translation through RPS6 and EIF4B. Mediates cellular proliferation, survival, and differentiation by modulating mTOR signaling and repressing pro-apoptotic function of BAD and DAPK1.	+	ND	ND	Ser741	Thrombin stimulates P90RSK that directly phosphorylates MAGI1 at Ser741 and induces MAGI1 deSUMOylation at Lys 931, leading to endothelial cells activation. This process involves Rap1 activation by phosphorylated MAGI1. SUMOylation impairs MAGI1 nuclear translocation and stabilizes endothelial barrier function.	[105,106]
*SENP2*	Sentrin/SUMO-Specific Protease SENP2	Cysteine protease targeting members of small ubiquitin-like modifier (SUMO) protein family. Deconjugates sumoylated proteins	+	ND	ND	Lys 931	MAGI1-K931 deSUMOylation induces both nuclear translocation of p90RSK-MAGI1 and ATF-6-MAGI1 complexes, which enhances endothelial cells activation and apoptosis, respectively.	[105]
**Viral (onco/) Proteins**								
**Molecular Partners**	**Virus**	**Viral Protein Function**	**MAGI1**	**Interaction** **MAGI2**	**MAGI3**	**MAGI Binding** **Domain**	**Biological** **Impact**	**References**
NS1	Influenza A virus. RNA virus	Non-structural (NS) protein; non-essential virulence factor. Involved in inhibition of host immune responses (IFN production/ response)	+	+	+	PDZ nd	Sequesters MAGI1 from the plasma membrane. Does not seem to confer benefit to viral replication	[107,108]
Tax	Human T-cell leukemia virus type 1 (HTLV-1). Retrovirus	Viral oncoprotein, immortalization of human T-cells. Promotes viral gene transcription and regulates expression of human genes by modulating CREB/ATF, NFkB, AP-1, and SRF pathways	+	*MAGI2* mRNA not detectable in T-cell lines	+	PDZ1	Downregulation of MAGI1 transcripts. Mis-localization of MAGI1, 3 to perinuclear region (from detergent-soluble to detergent-insoluble fraction). MAGI3 increases Tax1 accumulation	[109,110]
E4-ORF1	Human adenovirus type 9. DNA virus	Viral oncoprotein; PDZ binding motif required for E4-ORF1 -induced PI3K activation and cell transformation	+	ND	ND	PDZ1 and PDZ3	Ad9 E4-ORF1 protein sequesters MAGI1 in the cytoplasm (from detergent-soluble to detergent-insoluble fraction). Altered tight junction assembly and cell polarity	[111,112,113,114]
E6	Human papillomavirus (HPV); high-risk HPV (HPV 16 and 18) causes cervical cancer. DNA virus	Viral oncoprotein interacts with and promotes degradation of TP53 as well as proteins with PDZ domains, including MAGIs	+	+	+	PDZ1	HPV E6 proteins targets MAGI1 for proteasome degradation, leading to tight junction disruption. Restoration of MAGI1 expression in HPV positive cervix cancer HeLa cell line induces cell growth arrest and apoptosis. RNA aptamers to E6 inhibiting interaction with PDZ domain trigger cancer cell apoptosis	[111,115,116,117,118,119]
E protein	Coronavirus (MERS-CoV). RNA virus	The envelope protein forms a transmembrane ion channel. PDZ binding motifs of E proteins from MERS and SARS-CoV/SARS-COV-2 differs and interact with distinct and shared cellular proteins with the PDZ domain	+	+	ND	PDZ5		[120]

ND; nd: Not Determined. * In the literature, the PDZ domains of MAGIs are either numbered from 0 to 5 -considering the initially reported interactions of MAGI’s PDZ with their molecular partners and the lastly identified PDZ0-, or from 1 to 6 using an ordinal numbering. To avoid confusion, in the present review, we have normalized the numbering of the PDZ domains from 0 to 5.

**Table 2:** MAGI genetic alterations, silencing, posttranscriptional modifications in cancer.

Molecular Alterations	*MAGI*	Types of Cancer		Clinical Significance		References
**Promoter Methylation**						
	*MAGI1*	Anaplastic thyroid cancer		Malignant tumor associated with poor survival		[121]
		Lymphocytic leukemia		Decreased overall survival		[122]
	*MAGI2*	Breast cancer		Decreased overall survival		[128]
		Cervical cancer				[124]
		Colon cancer		32.8% exhibit MAGI2 hypermethylation		https://cancer.sanger.ac.uk/cosmic/ accessed on 22 July 2021
		Endometrium cancer		34.9% exhibit MAGI2 hypermethylation		[126]; https://cancer.sanger.ac.uk/cosmic/ accessed on 22 July 2021
		Gastric cancer		Decreased progression-free survival		[125]
		Ovarian cancer		Decreased overall survival		[126,127]
**Rearrangement**						
	*MAGI2*	Melanoma cell lines				[130]
		Prostate cancer				[131]
	*MAGI3*/*AKT3*	Breast cancer		Enriched in triple-negative breast cancers		[132]
**miRNA Targeting**						
miR-486-5p	*MAGI1*	Erythroid leukemia K562 cell line		Decreased erythroid differentiation		[136]
miR-520h		Renal cell carcinoma		Decreased overall survival		[137]
miR-629-5p	*MAGI2*	Ovarian cancer		miR-629-5p is downregulated in ovarian cancer		[138]
miR-27a-3p		Breast cancer/ macrophages		Exosomal miR-27a-3p from breast cancer exhausts MAGI2 in macrophages, leading to downregulation of PTEN, increased PDL1 expression, and cancer cells’ immune escape.		[142]
miR-487a		Breast cancer		miR-487a expression correlates with lymph node metastases		[139]
miR-101		Breast cell line (MCF7 cells)				[140]
miR-134/487b/655 cluster		Lung cancer cell lines		Epithelial–mesenchymal transition. Resistance to the EGFR inhibitor gefitinib,		[141]
miR-4677-3p		Glioma		The GLIDR lncRNA sponges miR-4677-3p that exhausts MAGI2. MAGI2 overexpression promotes glioma cell lines proliferation.		[144]
miR-34c-3p	*MAGI3*	Hepatocarcinoma		Decreased overall survival		[143]
**Premature Polyadenylation**						
	*MAGI3*	Breast cancer		Competition MAGI3pPa / wild-type MAGI3; activation YAP1 signaling		[42]
***MAGI1* Point Mutations**			***MAGI2* Point Mutations**		***MAGI3* Point Mutations**	
**Tissue**	% Mutated *		**Tissue**	% Mutated *	**Tissue**	% Mutated *
Liver	16.5		Prostate	26.4	Liver	9.8
Skin	13.6		Pancreas	23.5	Nervous system	9.8
Prostate	13.4		Liver	21.2	Skin	8.2
Pancreas	12.3		Breast	17.8	Prostate	7.7
Breast	10.8		Stomach	14.4	Biliary tract	6.7
Endometrium	10.1		Skin	14.4	Ovary	6.2
Nervous system	9.8		Esophagus	14.3	Breast	6.1
Ovary	9.5		Nervous system	13.3	Pancreas	5.9
Stomach	9.2		Ovary	13.2	Endometrium	5,8
Biliary tract	8.9		Endometrium	10.2	Esophagus	5.6

* Highest incidence of mutations in cancer, from Cosmic database.

**Table 3:** MAGI expression and cancer prognosis.

MAGI	Types of Cancer	Transcript Accumulation *	Protein Expression *	Clinical Significance	References
MAGI1	Breast cancer luminal	down	down	Decreased disease-free survival	[88]
	Breast cancer ER+/HER2−	down	down	High expression good prognosis	[147]
	Colon cancer	down	nd	Decreased disease-free survival	[161]
	Gastric cancer	down	down	Distant metastases	[146]
	Gliomas	down	down	Distant metastases	[56]
	Hepatocellular carcinoma	down	down	Poor prognosis	[145]
	Kidney			High RNA level, increased overall survival	[162] http://gepia2.cancer-pku.cn, accessed on 26 July 2021
MAGI2	Kidney			High RNA level, increased overall survival	[162] http://gepia2.cancer-pku.cn, accessed on 26 July 2021
	Multiple myeloma	up	nd	Decreased overall survival	[156]
	Ovarian cancer	down	nd	Decreased overall survival	[127]
	Prostate	down	unaffected/down	High mRNA levels better prognosis	[149,150,151]
		nd	up		[152,153,155]
MAGI3	Gliomas	down	down	Decreased overall survival	[55]
	Kidney			High RNA level, increased overall survival	[162] http://gepia2.cancer-pku.cn accessed on 26 July 2021
	Ovarian cancer	down	nd	Decreased disease-free survival	[157]
	Thyroid cancer	down	nd	Decreased RNA level in malignant vs benign tumors	[163]

* Transcript and protein levels in cancer compared with control tissue; nd: not determined.

**Table 4:** Molecular targets of MAGI ceRNAs and effectors systems. Physiopathological implications.

MAGI ceRNA		Primary Target		Secondary Target *	Activity, Function of Secondary Target	Physiological Impact	References
**MAGI1-IT1**							
MAGI1-IT1	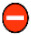	miR-200a	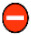	ZEB1, ZEB2 (zinc finger E-Box binding homeobox 1/ 2)	Transcriptional repressor. Promotes epithelial to mesenchymal transition	Epithelial–mesenchymal transition, invasiveness. Ovarian cancer cell lines	[206]
	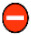	miR-302d-3p	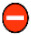	IGF1 (insulin-like growth factor 1)	Structurally and functionally related to insulin. Growth-promoting activity through binding to IGF1R tyrosine kinase, leading to activation of the PI3K-AKT/PKB and the Ras-MAPK pathways.	Overexpression MAGI1-IT1 in gastric cancer is associated with poor overall survival. Knockdown in gastric cancer cell lines decreases proliferation in vitro and in vivo	[207]
	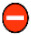	miR-512p-3p	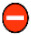	AKT (protein kinase B, PKB)	Proto-oncogene. Ser/Thr kinase downstream of phosphatidylinositol 3-kinase (PI3K). Involved in cell proliferation, survival, and migration.	Increased AKT activity. Increased lung cancer cell lines proliferation	[205]
**MAGI2-AS3 Tumor Suppressor Activity**							
MAGI2-AS3	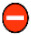	miR-15b-5p	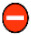	CCDC19/CFAP45 (cilia and flagella associated protein 45)	Tumor suppressor. Inhibits cell proliferation	Inhibition of proliferation, migration, and invasion. Bladder cancer cell lines	[208]
			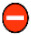	**RECK** * (reversion inducing cysteine-rich protein with kazal motifs; ST15, suppressor of tumorigenicity 15 protein)	Extracellular protein anchored to the plasma membrane. Decreases diffusible gelatinase/collagenase activity	Downregulation of miR15b-5p and miR-374a/b-5p by MAGI2-AS3 decrease the viability and migration of the epithelial ovarian cancer PEA1, KURAMOCHI, and SKOV3 cell lines. Downregulation of MAGI2-AS3 due to promoter hypermethylation	[209]
			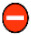	MTSS1 (metastasis suppressor protein 1)	Tumor suppressor controlling migration and invasion. Involved in Hedgehog and EGF pathways. Interacts with cytoskeleton	Downregulation of miR-15b-5p and miR-374a/b-5p by MAGI2-AS3 decrease the viability and migration of the epithelial ovarian cancer PEA1, KURAMOCHI, and SKOV3 cell lines. Downregulation of MAGI2-AS3 due to promoter hypermethylation	[209]
	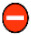	miR-23a-3p	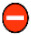	**PTEN** * (phosphatase and tensin homolog deleted on chromosome 10)	Lipid and protein phosphatase. Dephosphorylates PtdIns(3,4,5)P3 produced by PI3K. Inhibits AKT pathway. Dephosphorylates proteins, e.g., FAK, IRS1, Dvl2, PTEN. Exerts activity independent of its phosphatase activity, e.g., DNA damage repair. Induces apoptosis, decreases cell proliferation and migration	Decreased proliferation, migration, and invasion. Increased apoptosis. Lung squamous carcinoma cell lines	[210,211]
	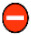	miR-25	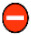	**RECK** * (reversion inducing cysteine-rich protein with kazal motifs; ST15, suppressor of tumorigenicity 15 protein)	Extracellular protein anchored to the plasma membrane. Decreases diffusible gelatinase/ collagenase activity	Decreased cell migration and invasiveness. Non-small cell lung carcinoma H1993 cell line	[212]
	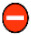	miR-31-5p	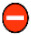	TNS1 (tensin 1)	Focal adhesion molecule, crosslinks actin filaments. Involved in cell migration	Decreased proliferation, migration, and invasion of bladder cancer cell lines	[213]
	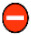	miR-155	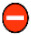	SOCS1 (suppressor of cytokine signaling 1)	Negative regulation of cytokines signaling through the JAK/STAT3 pathway	Decreased cell proliferation. Non-small cell lung carcinoma H1993 cell line	[214]
	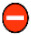	miR-223-3p (according to the sequence in Ref. [215])	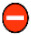	EBP41L3 (erythrocyte membrane protein band 4.1 Like 3)	Tumor suppressor. Member of the band 4.1 family of cytoskeletal proteins. The linker between membrane proteins and cytoskeleton. Involved in cell adhesion and motility	Inhibition of the human cervix cancer SiHa and HeLa cell lines invasion and migration	[215]
	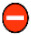	miR374a/b-5p	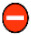	CADM2 (cell adhesion molecule 2)	Transmembrane protein involved in cell aggregation	Decreased proliferation and migration in vitro, enhanced apoptosis, decreased tumor growth, and experimental metastases in nude mice. Lung squamous carcinoma xxxSW900, SK-MES_1, and A549 and cell line.	[211]
			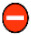	**PTEN** * (phosphatase and tensin homolog deleted on chromosome 10)	Lipid and protein phosphatase. Dephosphorylates PtdIns(3,4,5)P3 produced by PI3K. Inhibits AKT pathway. Dephosphorylates proteins, e.g., FAK, IRS1, Dvl2, PTEN. Exerts activity independent of its phosphatase activity, e.g., DNA damage repair. Induces apoptosis, decreases cell proliferation and migration	Downregulation of miR15b-5p and miR-374a/b-5p by MAGI2-AS3 decrease the viability and migration of the epithelial ovarian cancer PEA1, KURAMOCHI, and SKOV3 cell lines. Downregulation of MAGI2-AS3 due to promoter hypermethylation. Decreased migration and invasion of breast cancer cell lines	[209,216]
	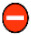	miR374b-5p	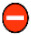	SMG1 (genitalia family member 1)	Ser/Thr protein kinase involved in nonsense-mediated decay of mRNAs containing premature Stop codons. Can phosphorylate and trigger TP53 activation after cell exposure to genotoxic stress	Decreased proliferation and migration. Hepatocarcinoma cell lines	[217]
			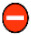	**RECK** * (reversion inducing cysteine-rich protein with kazal motifs; ST15, suppressor of tumorigenicity 15 protein)	Extracellular protein anchored to the plasma membrane. Decreases diffusible gelatinase/collagenase activity	Downregulation of miR15b-5p and miR-374a/b-5p by MAGI2-AS3 decrease the viability and migration of the epithelial ovarian cancer PEA1, KURAMOCHI, and SKOV3 cell lines. Downregulation of MAGI2-AS3 due to promoter hypermethylation	[209]
			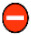	HOXA5 (homeobox A5)	Transcription factor upregulates the tumor suppressor p53	Downregulation of miR15b-5p and miR-374a/b-5p by MAGI2-AS3 decrease the viability and migration of the epithelial ovarian cancer PEA1, KURAMOCHI, and SKOV3 cell lines. Downregulation of MAGI2-AS3 due to promoter hypermethylation	[209]
	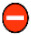	miR-525-5p	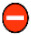	MDX1 (MAX dimerization protein)	Competes with MAX (Myc associated factor X) for interaction with the c-Myc proto-oncogene. Inhibits transactivation by this transcriptional activator complex	Decreases proliferation and migration of ovarian cancer cell lines	[218]
	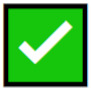	EZH2 (Enhancer of zeste homolog 2; histone-lysine N-methyltransferase)	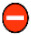	HOXB7 (homeobox B7)	Member of the homeobox family of transcription factors. Involved in cell proliferation and differentiation. Master regulatory gene orchestrating several oncogenic pathways. Overexpressed in various types of cancers including breast, esophageal, gastric, ovarian, and melanoma	MAGI2-AS3 binds to the *HOXB7* promoter and recruits EZH2. Methylation of lysine 27 on histone H3 in the promoter region negatively regulates the transcription of *HOXB7* and inhibits proliferation and radio-resistance of esophageal cancer cells.	[219]
	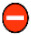	HEY1 (Hes related Family BHLH transcription factor with YRPW motif 1)	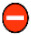	ACY1 (aminoacylase 1)	Involved in the hydrolysis of various N-acetylated amino acids. Putative tumor suppressor in clear cell renal cell carcinoma	MAGI2-AS3 forms a nucleoprotein complex with HEY1 in the nucleus and impairs its transcriptional repressor activity on *the ACY1* promoter. Decreased cell viability, EMT, and migration in vitro; reduced tumor growth and angiogenesis in vivo. Clear cell renal cell carcinoma	[220]
	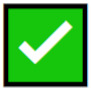	KDM1A (lysine demethylase 1A	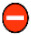	RacGAP (Rac GTPase activating protein 1)	Negative regulation of Rho GTPase signaling through stimulation of GTP hydrolysis	Demethylation of Lys4 of histone H3 (H3K4Me2) at *RACGAP1* promoter reduces transcription. Decreased proliferation and migration, enhanced apoptosis of HEPG2 hepatocarcinoma cells in vitro, and decreased tumor growth in nude mice	[221]
	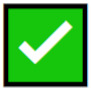	TET1 (Tet Methylcytosine Dioxygenase 1)	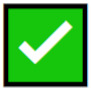	MAGI2 (membrane-associated guanylate kinase inverted 2)	Scaffolding molecule with 1 Guanylate Kinase domain, 2 WW domains, and 6 PDZ Domains. Involved in junctional complexes stability and cell signaling	Demethylation of *MAGI2* promoter, increased MAGI2 expression, inhibition of AKT and Wnt/ β-catenin pathways; decreased cell proliferation of MCF-7 breast cancer cell line	[128]
	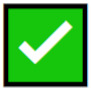	TET2 (Tet Methylcytosine Dioxygenase 2)	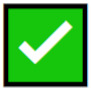	LRIG1 (leucine-rich repeats and immunoglobulin-like domains 1)	Transmembrane protein that interacts and negatively regulates receptor tyrosine kinases of the EGFR family, MET and RET by enhancing receptor ubiquitination and degradation	Impaired leukemia stem cells self-renewal	[222]
	?	?	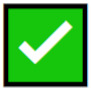	FAS (Fas cell surface death receptor); FASL (FAS ligand)	Members of the TNF/ TNF receptor family. Promotes activation of caspase-8 and triggers the apoptotic cascade	Decreased proliferation, enhanced apoptosis of MDA-MB-231 and MCF-7 breast cancer cell lines. The underlying mechanism has not been characterized but might involve miR-374a -a target of MAGI2-AS3- that exhausts FAS transcripts	[223]
	?	?	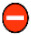	ROCK2 (Rho-associated coiled-coil containing protein kinase 2)	Ser/Thr kinase, a downstream effector of the Rho GTPase. Involved in the regulation of actin cytoskeleton organization, stress fiber and focal adhesion formation, cell adhesion, and motility	MAGI2-AS3 induces the downregulation of ROCK2 at both RNA and protein levels in the Hep3B and MHCC97-H hepatocarcinoma cell lines decrease cell migration and invasiveness and induce apoptosis.	[224]
**MAGI2-AS3 Tumor Promoter Activity**							
MAGI2-AS3	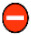	miR-141	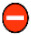	**ZEB1** * (zinc finger E-box binding homeobox 1)	Transcriptional repressor. Promotes epithelial to mesenchymal transition	Increased migration and invasion of gastric cancer cell lines	[225]
	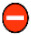	miR-143	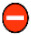	AKT, COX2, ERK5, HMBG1, IGF1-R, PAI-1 ?		MiR-143 functions as a tumor suppressor in human bladder cancers. Known targets of miR-143 in bladder cancers cells are AKT, Cox2, ERK5, HMBG1, IGF1-R, PAI-1	[226]
	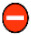	miR-200a	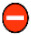	**ZEB1** *, ZEB2 (zinc finger E-box binding homeobox 1/ 2)	Transcriptional repressor. Promotes epithelial to mesenchymal transition	Increased migration and invasion of gastric cancer cell lines. The bromodomain-containing 4 (BRD4) protein, an acetylated histone binding protein is a transcriptional activator of MAGI2-AS3 in gastric cancer. MiR-200a increases E-cadherin levels, inhibits migration, and enhances the sensitivity of bladder cancer cell lines to anti-EGFR therapies.	[225,226,227]
	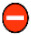	miR-218-5p	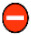	GDPD5 (glycerophosphodiester phosphodiesterase domain containing 5)	Involved in glycerol metabolism. Mediates the cleavage of glycosylphosphatidylinositol -anchor of RECK, leading to RECK release from the plasma membrane	Increased proliferation, migration, epithelial–mesenchymal transition, and cisplatin resistance of nasopharyngeal epithelial cell lines	[228]
	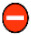	miR-3163	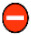	TMEM106B (transmembrane protein 106B)	Involved in the regulation of lysosomal trafficking	Decreased apoptosis, increased proliferation, and migration of the HCT116 and RKO colon cancer cell lines	[229]

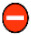
: Inhibition of the target; 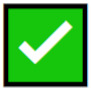: Activation of the target; * Secondary targets regulated by different miRNAs exhausted by MAGI2-AS3 are in bold.
